# CsCu_2_I_3_ Nanoparticles Incorporated within a Mesoporous Metal–Organic Porphyrin Framework as a Catalyst for One-Pot Click Cycloaddition and Oxidation/Knoevenagel Tandem Reaction

**DOI:** 10.1021/acsami.2c04364

**Published:** 2022-08-08

**Authors:** Saba Daliran, Mostafa Khajeh, Ali Reza Oveisi, Josep Albero, Hermenegildo García

**Affiliations:** † Department of Chemistry, University of Zabol, P.O. Box 98615-538, Zabol 98615-538, Iran; ‡ Departamento de Química and Instituto de Tecnología Química CSIC-UPV, Universitat Politècnica de València, Av. de los Naranjos s/n, 46022 Valencia, Spain

**Keywords:** heterogeneous catalysis, metal−organic frameworks as hosts, CsCu_2_I_3_ perovskite, multifunctional catalyst, photo-oxidation

## Abstract

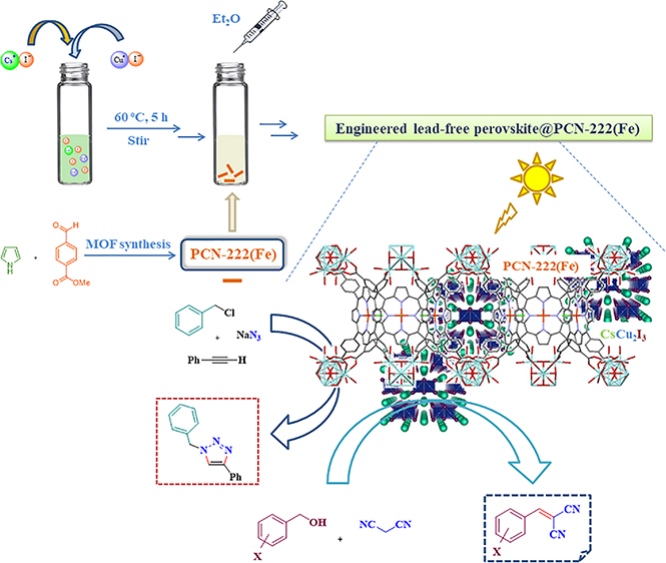

Metal–organic frameworks (MOFs) and metal halide perovskites are currently under much investigation due to their unique properties and applications. Herein, an innovative strategy has been developed combining an iron-porphyrin MOF, PCN-222(Fe), and an *in situ*-grown CsCu_2_I_3_ nontoxic lead-free halide perovskite based on an earth-abundant metal that becomes incorporated within the MOF channels [CsCu_2_I_3_@PCN-222(Fe)]. Encapsulation was designed to decrease and control the particle size and increase the stability of CsCu_2_I_3_. The hybrid materials were characterized by various techniques including FE-SEM, elemental mapping and line scanning EDX, TEM, PXRD, UV–Vis DRS, BET surface area, XPS, and photoemission measurements. Hybrid CsCu_2_I_3_@PCN-222(Fe) materials were examined as heterogeneous multifunctional (photo)catalysts for copper(I)-catalyzed alkyne-azide cycloaddition (CuAAC) and one-pot selective photo-oxidation/Knoevenagel condensation cascade reaction. Interestingly, CsCu_2_I_3_@PCN-222(Fe) outperforms not only its individual components CsCu_2_I_3_ and PCN-222(Fe) but also other reported (photo)catalysts for these transformations. This is attributed to cooperation and synergistic effects of the PCN-222(Fe) host and CsCu_2_I_3_ nanocrystals. To understand the catalytic and photocatalytic mechanisms, control and inhibition experiments, electron paramagnetic resonance (EPR) measurements, and time-resolved phosphorescence were performed, revealing the main role of active species of Cu(I) in the click reaction and the superoxide ion (O_2_^•–^) and singlet oxygen (^1^O_2_) in the photocatalytic reaction.

## 1 Introduction

Perovskites, as semiconductors, are prospective materials for emerging optoelectronic applications owing to their remarkable physical and chemical features, low manufacturing cost, and adjustable tenability.^1,2^ Lead-based perovskites, as the most common perovskites, can be highly efficient for light harvesting; however, they exhibit very low stability and high toxicity, which limit their practical applications.^3,4^ Therefore, intensive research has been conducted to develop alternative low toxicity and efficient lead-free perovskites. Among the newly studied lead-free candidates, copper-based perovskites are among the most promising. Particularly, 1D CsCu_2_I_3_ exhibits a decent photoresponse and lesser toxicity as well as being constituted by the earth-abundant Cu element.^1,5−8^ However, there are some critical points that hamper Cu perovskite preparation, such as the need to add multiple capping agents to limit crystal growth and to stabilize the structure, the use of DMF as a solvent, and/or long crystal growth time. Furthermore, due to their lack of structural stability, the activity of Cu perovskites in (photo)catalysis is largely unexplored, which is important to gain information of their properties as catalysts.

Typically, instable perovskites, such as lead halide perovskites, are stabilized on silica/alumina^9,10^ or polymer supports,^11^ but frequently, the perovskite is not homogeneously distributed, there are substrate diffusion limitations, and there is metal leaching to the liquid phase. One alternative strategy is to use periodic, well-defined metal–organic frameworks (MOFs) as supports.^12−15^ Metal–organic frameworks (MOFs), formed by inorganic nodes and organic connectors interacting by Coulombic and coordination bonds, are highly crystalline and well-defined unique porous frameworks with variable structures and chemical functionalities, tunable pore sizes, high specific surface areas, large internal pore volumes, and low framework densities.^16,17^ Because of these intrinsic features, MOFs have been massively studied in various areas.^17−22^

Research has shown that hybridization of perovskites with MOFs results in the formation of new multicomponent materials with enhanced stability, higher efficiency, and improved charge transfer and performance compared to the individual components^12−15^ or classical supports.^9−11^ However, most of reported MOF supports possess micropores (<2 nm), which restricted the mass diffusion and perovskite growth and affected the stability of the MOF structure.^14,15^ Therefore, to address these issues, MOFs with ordered mesopores and high porosity, maintaining their stability during and after perovskite loading, are highly demanded but challenging. Particularly, the stabilization of 0D or 1D perovskites provided by encapsulation within MOFs can enable evaluation of the catalytic activity of occluded halide perovskites, something that has remained elusive so far. In addition, the MOF can cooperate with the activity of the occluded perovskite, producing a synergy between the two components in a multifunctional material.

PCN-222(Fe) is a distinctive metalloporphyrin MOF with a high surface area involving Zr_6_ clusters attached to Fe-TCPP (TCPP = 4,4′,4″,4‴-(porphine-5,10,15,20-tetrayl)tetrakis(benzoic acid)) linkers. In addition, PCN-222(Fe) possesses regular hexagonal meso- and triangular microporous channels and high thermal and chemical stability,^23−26^ inside which CsCu_2_I_3_ can be accommodated.

Herein, it is shown that encapsulation of the CsCu_2_I_3_ perovskite inside a mesoporous iron-porphyrin PCN-222(Fe) MOF as a host can be achieved by a facile *in situ* growth to render CsCu_2_I_3_@PCN-222(Fe). By varying the synthetic parameters, CsCu_2_I_3_ units uniformly distributed in PCN-222(Fe) and having a strong interaction with the PCN-222(Fe) framework can be prepared. PCN-222(Fe) functions as a support, stabilizing and confining the size and shape of the encapsulated CsCu_2_I_3_ nanostructures. Stabilization provided by PCN-222(Fe) has allowed the study of the activity of CsCu_2_I_3_ as a heterogeneous catalyst for a three-component CuAAC reaction and one-pot cascade photo-oxidation of benzyl alcohols/Knoevenagel coupling reaction. It was observed that CsCu_2_I_3_@PCN-222(Fe) exhibits high activity for these reactions, outperforming the individual components and other reported catalysts. Remarkably, CsCu_2_I_3_@PCN-222(Fe) was stable and could be easily recovered from the reaction media for reuse with no obvious decay in activity. This stability is remarkable considering the notorious instability characteristic of halide perovskites. Mechanistic studies indicated a synergistic cooperation between CsCu_2_I_3_ and PCN-222(Fe). In addition, the host–guest system was further confirmed by using various techniques as described here. The design of multifunctional solid catalysts based on nontoxic elements and the characterization of the synergistic effects derived from the encapsulation of CsCu_2_I_3_, well studied in the field of photovoltaics but not in catalysis, are two of the current research fronts in advanced materials synthesis.

## 2 Experimental Section

### 2.1 Materials

Chemical materials such as pyrrole, methyl benzaldehyde-4-carboxylate, propionic acid, benzoic acid, zirconium(IV) chloride, iron dichloride tetrahydrate, *N*,*N*′-diethylformamide (DEF), cesium iodide, copper(I) iodide, acetonitrile, sodium azide, benzyl chloride, phenylacetylene, malononitrile, and benzyl alcohols were synthetically pure and were supplied by Merck. The Fe-TCPP linker was synthesized following a procedure described in the literature.^23^ PCN-222(Fe) was synthesized and activated with the HCl-treatment procedure.^23^ Synthesis of pure CsCu_2_I_3_ perovskite was performed by a modified procedure reported in the literature^7^ as mentioned here.

### 2.2 Characterization

High-quality X-ray diffraction patterns were acquired with Cu K_α1_ radiation on a Philips, X’Pert XRD diffractometer (Netherlands). FE-SEM imaging, energy dispersive X-ray spectroscopy (EDX), and elemental mapping measurements were performed on a scanning electron microscope (TESCAN MIRA3, Czech Republic). Thermogravimetric analysis (TGA) tests were conducted on a TGA/DSC instrument (Mettler Toledo, Germany) under an inert atmosphere. Nitrogen adsorption/desorption isotherms at −196 °C were obtained on an ASAP apparatus (Micromeritics, USA). The NMR spectra of the products were recorded on a Bruker Avance DPX-250 NMR (300 MHz for ^1^H NMR and 75 MHz for ^13^C NMR) in deuterated solvents. The diffuse reflectance spectra were collected by using ultraviolet–visible diffuse reflectance spectroscopy (UV–Vis DRS, Shimadzu Co., Japan). The visible exposure was achieved by 280 power light-emitting diode (LED) lamps (3.2 V, 1 W) in a cylindrical container, 32,000 LUX, including air cooling fans to keep the temperature constant at 31–35 °C. The X-ray photoelectron spectra (XPS) were measured on a SPECS spectrometer equipped with a Phoibos 150 9MCD detector using a nonmonochromatic X-ray source (Al) operating at 200 W. The samples were evacuated in the prechamber of the spectrometer at 1 × 10^–9^ mbar. The measured intensity ratios of the components were obtained from the area of the corresponding peaks after nonlinear Shirley-type background subtraction and corrected by the transition function of the spectrometer. The work function of the apparatus was calibrated with Ag, Au, and Cu with a value of 4.2440 eV. The EPR spectra were recorded using a Bruker EMX, with the typical settings: frequency, 9; 80 GHz; sweep width, 100 G; time constant, 80 ms; modulation frequency, 100 kHz; modulation width, 0.2 G. Singlet oxygen was studied by time-resolved near-infrared phosphorescence using a home-made setup. Briefly, a pulsed Nd:YAG laser (FTSS355-Q, Crystal Laser) working at 1 kHz repetition rate at 355 nm (third harmonic; 0.5 μJ per pulse) was used for sample excitation. A 1064 nm Rugate notch filter (Edmund Optics) and an uncoated SKG-5 filter (CVI Laser Corporation) were placed at the exit port of the laser to remove any residual component of its fundamental emission in the near-infrared region. The luminescence exiting from the sample was filtered by a 1100 nm long-pass filter (Edmund Optics) and a narrow bandpass filter at 1275 nm (BK-1270-70-B, bk Interferenzoptik). A thermoelectric-cooled near-infrared sensitive photomultiplier tube assembly (H9170-45, Hamamatsu Photonics) was used as a detector. High-resolution TEM (HRTEM) images, scanning TEM (STEM) images, and energy-dispersive X-ray (EDX) spectra were recorded by using a JEOL, JEM 2100F microscope. Steady-state fluorescence measurements were carried out using a Photon Technology International (PTI, Germany) LPS-220B spectrofluorometer equipped with a monochromator in the range of 200–800 nm. The excitation wavelength was 360 nm and emission was recorded from 390 to 700 nm by 1 nm steps with an integration time of 0.1 s, averaging three measurements.

### 2.3 Synthesis of CsCu_2_I_3_

CuI (1.3 mmol) and CsI (0.65 mmol) were added to a glass vial containing anhydrous acetonitrile (4 mL) before stirring at 60 °C for 5 h. Then, the vial was allowed to cool to room temperature and the saturated solution was filtered through a polytetrafluoroethylene (PTFE) (0.45 μm) filter adapted to a syringe. After that, diethyl ether (∼2 mL) was added dropwise to the filtered solution. The white precipitate of CsCu_2_I_3_ formed was allowed to grow for 15 min or 3.5 h and collected by centrifugation.

### 2.4 CsCu_2_I_3_ Confined into PCN-222(Fe)

CsCu_2_I_3_@PCN-222(Fe) nanostructures were fabricated by controlling the reaction time and temperature using the so-called antisolvent/inverse solvent infiltration approach. Briefly, CuI (1.3 mmol) and CsI (0.65 mmol) were sonicated in acetonitrile (4 mL) and stirred magnetically for 5 h at 60 °C. The mixture was cooled to ambient temperature, filtered through a polytetrafluoroethylene (PTFE) (0.45 μm) syringe, and further injected into glass vials containing activated PCN-222(Fe) (50 mg) before being capped and kept in ambient temperature or 60 °C for 16–18 h. After refreshing the top of the solvent, the CsCu_2_I_3_ crystals were allowed to grow at room temperature by dropwise addition of diethyl ether as an antisolvent (∼2 mL). As-synthesized CsCu_2_I_3_@PCN-222(Fe) materials with different amounts of CsCu_2_I_3_ were then washed several times with acetonitrile and acetone to give CsCu_2_I_3_@PCN-222(Fe) hybrid materials, here namely, 15 min-r.t., 3.5 h-r.t., 15 min-60 °C, and 3.5 h-60 °C.

### 2.5 One-Pot Three-Component Click Reaction

In an unsealed vial, benzyl chloride (0.5 mmol), sodium azide (1 mmol), and phenyl acetylene (1 mmol) were mixed in H_2_O (2 mL) and then the catalyst (10 mg) was added. The resulting mixture was stirred at room temperature and periodically checked by TLC (*n*-hexane/ethyl acetate (4:1)). After the completion of the reaction, the mixture was diluted by additional H_2_O (4 mL), and the catalyst was removed by centrifugation and washed with ethyl acetate. The organic layer was extracted three times with ethyl acetate, washed with saturated NaHCO_3_, and passed over anhydrous Na_2_SO_4_. Recrystallization from a mixture of ethyl acetate and hexane afforded the pure product.

### 2.6 Tandem Photo-oxidation/Knoevenagel Condensation Reaction

Into a test tube, benzyl alcohol (0.5 mmol), malononitrile (0.75 mmol), acetonitrile (2–3 mL), and catalyst (15 mg) were mixed and then the mixture was stirred at room temperature under exposure to visible LED illumination and oxygen (an O_2_-filled balloon). The temperature in the illuminated photoreactor was maintained at 33 ± 2 °C. After completion of the reaction, the mixture was checked by TLC (*n*-hexane/ethyl acetate (5:1)), the catalyst was extracted by centrifugation, and the solvent was evaporated under vacuum. The product was finally purified by recrystallization from ethanol/water.

## 3 Results and Discussion

### 3.1 Synthesis and Characterization of CsCu_2_I_3_@PCN-222(Fe)

**Figure 1:**
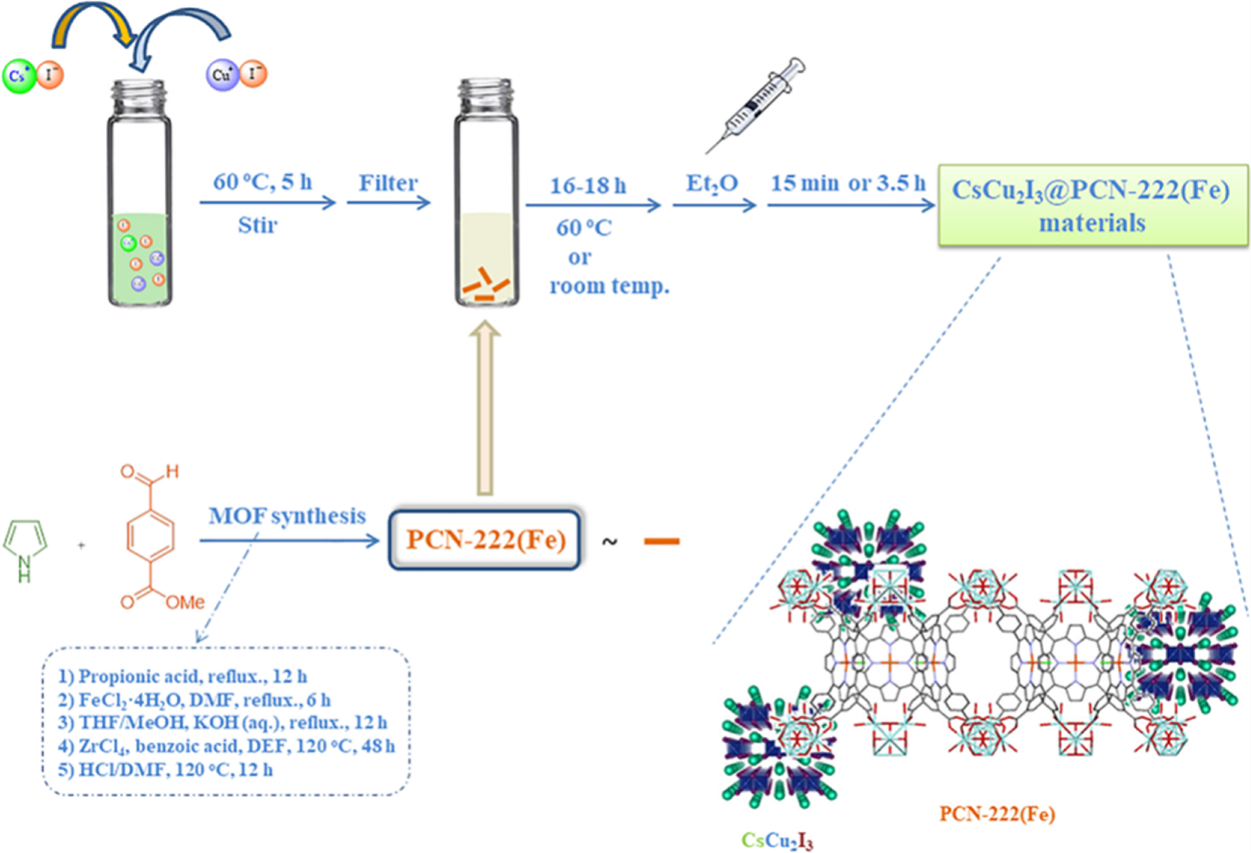
Synthesis route used for the preparation of CsCu_2_I_3_@PCN-222(Fe) hybrid materials.

CsCu_2_I_3_@PCN-222(Fe) samples with different porosity, sizes, pore structures, surface areas, and morphology were fabricated by controlling the reaction time and temperature employing an antisolvent/inverse solvent infiltration approach using the MOF support (after its synthesis) and CsCu_2_I_3_ precursors as shown in Figure 1 (see Section 2 for details). The use of an Fe-porphyrin avoids subsequent partial metalation of free-base porphyrin during the synthesis of halide perovskite while shortening the bandgap of PCN-222 and ensuring a strong O_2_ adsorption.

**Figure 2:**
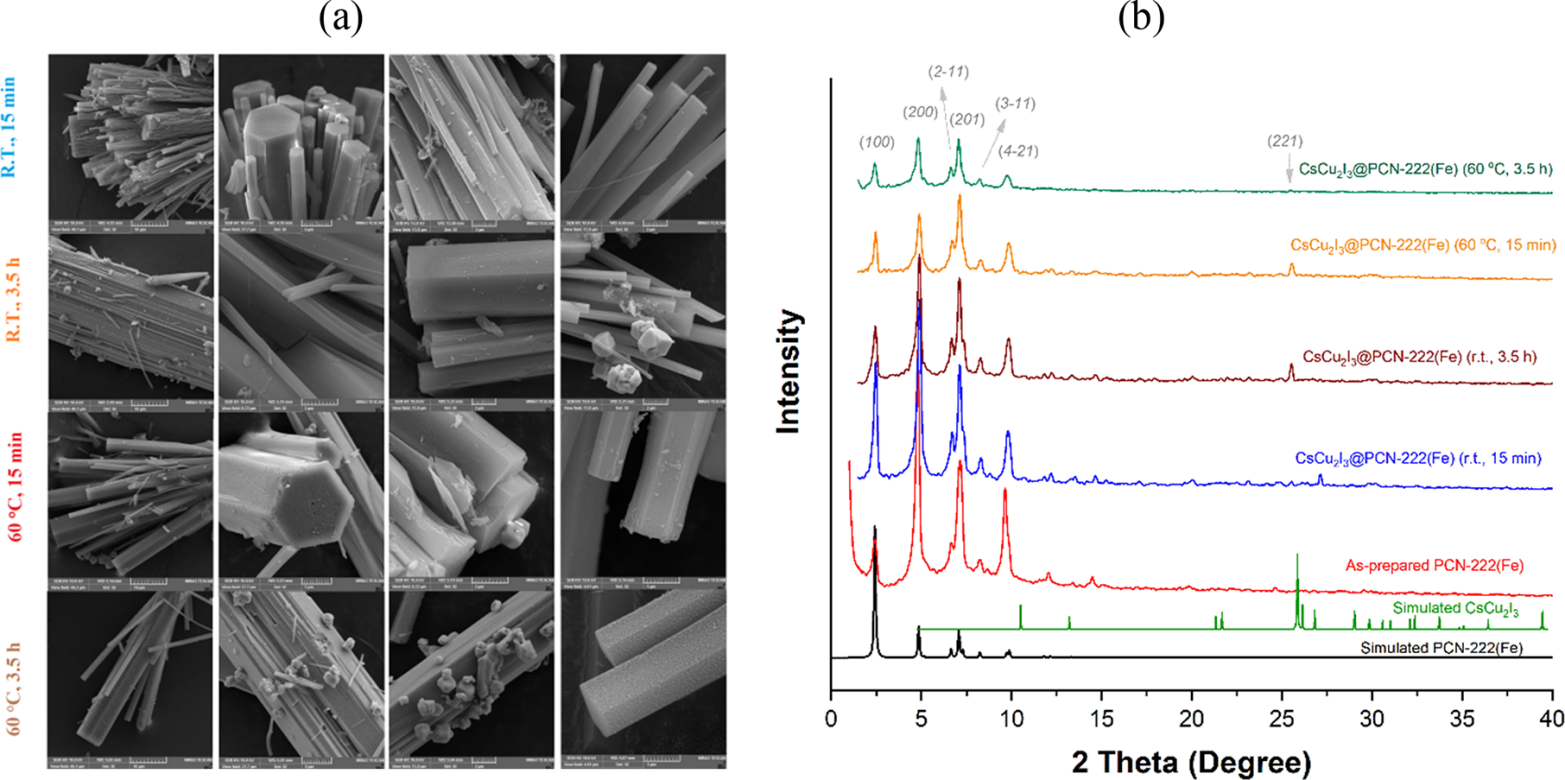
(a) SEM images at four different magnifications (from left to right: 10, 5, 2, and 1 μm) of as-synthesized CsCu_2_I_3_@PCN-222(Fe) hybrid materials under the different conditions (15 min-r.t., 3.5 h-r.t., 15 min-60 °C, and 3.5 h-60 °C) and (b) XRD patterns of simulated MOF, simulated CsCu_2_I_3_, and as-prepared MOF and CsCu_2_I_3_@MOF hybrid materials obtained at different times and temperatures (15 min-r.t., 3.5 h-r.t., 15 min-60 °C, and 3.5 h-60 °C).

Field emission scanning electron microscopy (FE-SEM) images of as-synthesized CsCu_2_I_3_@PCN-222(Fe) (15 min-r.t., 3.5 h-r.t., 15 min-60 °C, and 3.5 h-60 °C, denoting the time and temperature of the PCN-222 infiltration treatment), individual CsCu_2_I_3_, and pristine PCN-222(Fe) are shown in Figure 2a and Figure S1.

At a short time (15 min), the images clearly display the selective growth of CsCu_2_I_3_ with rod-like morphology along the *c* axis of hexagonal rod-shaped PCN-222(Fe) crystals. In addition, when the antisolvent was added and aged for 3.5 h, CsCu_2_I_3_ nanocrystals were further grown with irregular octahedron-like morphology especially apparent on the surface. It was obvious that the presence of the MOF takes control the growth rate and crystal size of CsCu_2_I_3_, effectively avoiding the agglomeration of the growing crystals (see Figure S1*versus*Figure 2a). As shown in the SEM images of Figure 2a and Figure S1, under otherwise identical conditions, smaller CsCu_2_I_3_ crystals are obtained in the presence of PCN-222(Fe) owing to its well-defined ordered porous structure, while bigger and enlarged CsCu_2_I_3_ crystals or large agglomerations are fabricated in the absence of the MOF. Furthermore, the results showed that the reaction time had more significant influence on the CsCu_2_I_3_ particle size and morphology than the reaction temperature.

Elemental composition of the synthesized samples determined by energy-dispersive X-ray spectroscopy (EDX) confirmed the presence of all the expected elements, namely, C, N, O, Fe, Cu, Zr, I, and Cs, showing the relative Cu/Cs molar ratios of 1.73, 1.99, 2.33, and 1.91, respectively, for the 15 min-r.t., 3.5 h-r.t., 15 min-60 °C, and 3.5 h-60 °C CsCu_2_I_3_@PCN-222(Fe) samples (Table S1), in fair agreement with the expected values. Furthermore, EDX elemental mapping of the structures indicated the uniform distribution of all the constituents (C, N, O, Fe, Cu, Zr, I, and Cs) over the CsCu_2_I_3_@PCN-222(Fe) crystals (Table S2).

The crystalline structures and successful synthesis of the CsCu_2_I_3_@PCN-222(Fe) hybrid materials were further proven by X-ray diffraction (XRD) patterns (Figure 2b), which evidently exhibited the diffraction peaks of PCN-222(Fe), whose main peaks at 2θ of 2.5°, 4.8°, 6.6°, 7.1°, 8.2°, and 9.8° correspond to diffraction through the (100), (200), (2-11), (201), (3-13), and (4-21) planes (CCDC no. 893545), respectively,^23^ and CsCu_2_I_3_, whose main diffraction peak at 2θ of 26.1° corresponds to diffraction in the (221) plane (PDF #45-0076). The PXRD patterns indicated that the growth of CsCu_2_I_3_ crystals had no effect on the PCN-222(Fe) structure. The experimental PXRD data of as-synthesized CsCu_2_I_3_ particles match well with the standard pattern of CsCu_2_I_3_ (Figure S2).^27,28^ The peaks at 2θ = 10.8°, 13.5°, 21.6°, 21.9°, 26.1°, 27.2°, 29.3°, 33.6°, 32.6°, and 39.8° can be ascribed respectively to the reflections from the (110), (020), (220), (130), (221), (040), (002), (202), (330), and (421) crystal planes of the orthorhombic phase. No difference in the XRD pattern of CsCu_2_I_3_ for the rod- or octahedron-like particles was observed.

**Figure 3:**
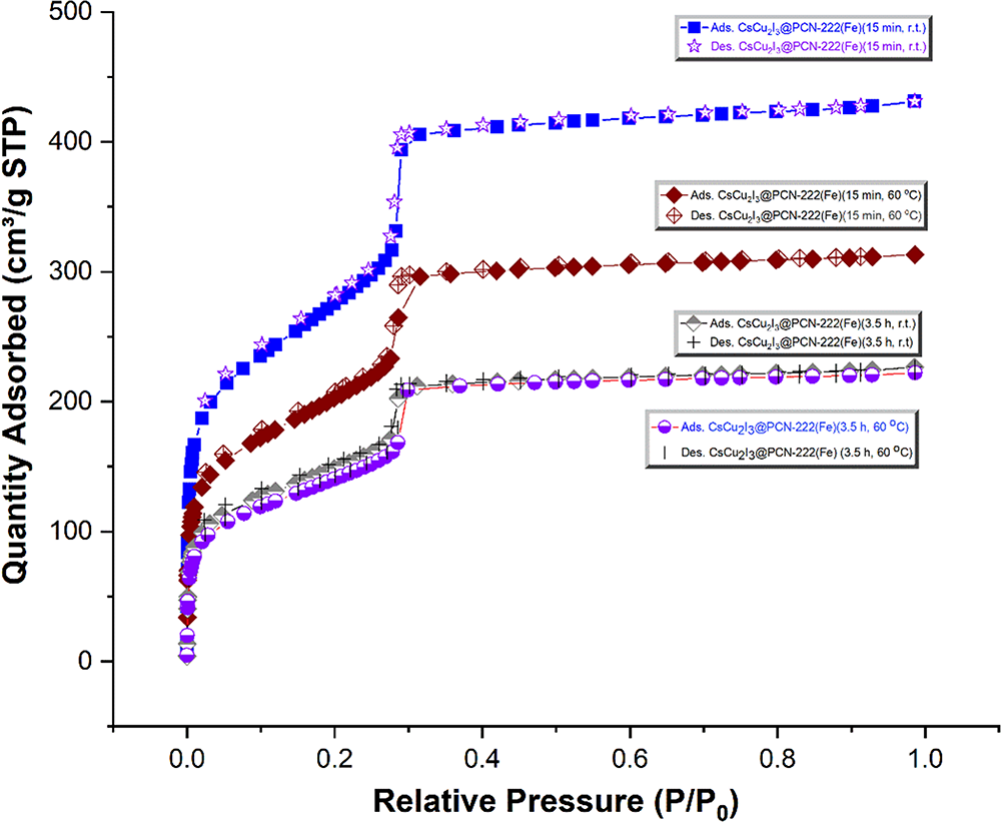
N_2_ adsorption–desorption isotherms for CsCu_2_I_3_@PCN-222(Fe) hybrid materials at 77 K (from top to bottom:15 min-r.t., 15 min-60 °C, 3.5 h-r.t., and 3.5 h-60 °C).

To investigate the permanent porosity of the samples, isothermal N_2_ adsorption–desorption measurements at 77 K were performed (Figure 3 and Figure S3). The N_2_ isotherms show a distinctive IUPAC type IV shape, revealing the existence of mesopores (a steep rise at about *P/P*_0_*=* 0.3) for all of them. The measured Brunauer–Emmett–Teller (BET) surface areas for PCN-222(Fe) and CsCu_2_I_3_@PCN-222(Fe) samples of 15 min-r.t., 3.5 h-r.t., 15 min-60 °C, and 3.5 h-60 °C were calculated to be 1715, 930, 500, 682, and 480 m^2^ g^–1^, respectively (Figure 3 and Figure S3). The observed decreases in BET surface areas are attributed to the *in situ* growth of CsCu_2_I_3_ into the PCN-222(Fe) MOF voids at different loadings and the pore-filling effect, in good agreement with the information provided by SEM/EDX data.

The porosity of PCN-222(Fe) and CsCu_2_I_3_@PCN-222(Fe) structures with tunable pores was further confirmed by using nonlocal density functional theory models to determine pore size distributions (PSDs). As a result, the pore diameters of PCN-222(Fe) after the *in situ* growth and immobilization of the CsCu_2_I_3_ perovskite decreased as the reaction time and temperature increased, proving further the successful synthetic steps (see Figures S3 and S4 in detail).

The UV–Vis DRS spectra of CsCu_2_I_3_@PCN-222(Fe) samples containing different amounts of CsCu_2_I_3_ perovskite recorded at room temperature are shown in Figure S5. Comparison of the UV–Vis DRS spectra of CsCu_2_I_3_@PCN-222(Fe) with those of PCN-222(Fe) shows, as the most remarkable feature, the appearance of an intense peak around 200–400 nm, which can be attributed to the CsCu_2_I_3_ perovskite, and notably, the absorbance of CsCu_2_I_3_@PCN-222(Fe) samples covered the entire UV–Vis area. In addition, these UV–Vis DRS spectra indicate that there is a strong interaction between PCN-222(Fe) and the CsCu_2_I_3_ guest, leading to changes in the measured spectra of the hybrid material with respect to the sum of the two components. The observed differences in the UV–Vis DRS spectra of the host–guest hybrids might be because of the different loadings of the CsCu_2_I_3_ perovskite. Furthermore, the optical bandgaps (*E*_g_) were estimated from the plot of (α*h*ν)^1/2^*versus* photon energy (*h*ν) (the Tauc equation) obtained from the diffuse reflectance data (Figure S6). As seen in Figure S6, the *E*_g_ values of CsCu_2_I_3_@PCN-222(Fe) samples estimated from the intercept of tangents to the Tauc plots were between ∼1.54 and 1.59 eV. Notably, these values are lower than that of the CsCu_2_I_3_ perovskite (∼3.78 eV).^1,5,7,8^

**Figure 4:**
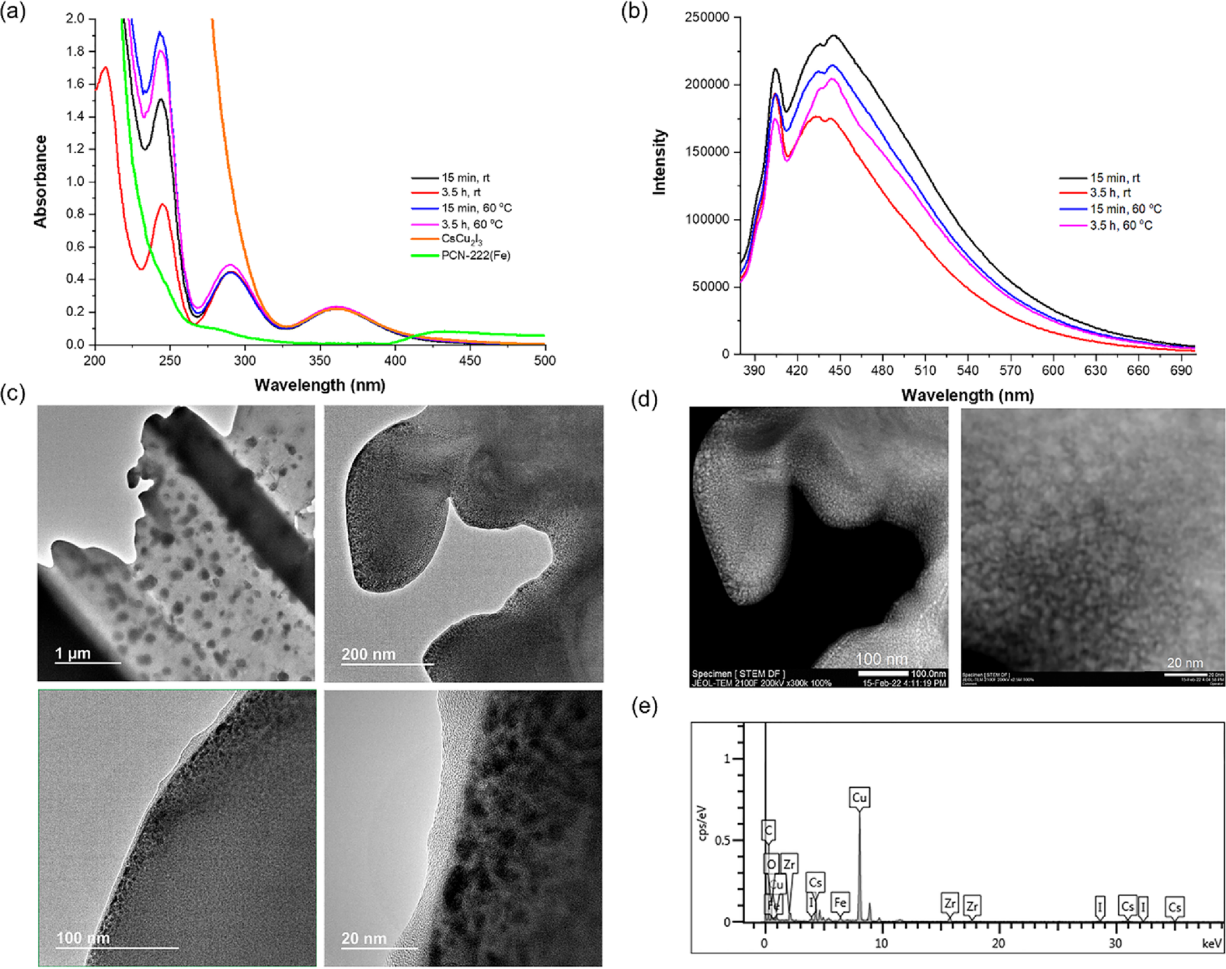
(a) Steady-state UV–Vis extinction, (b) emission spectra of CsCu_2_I_3_@PCN-222(Fe) composites (λ_exc_ = 360 nm), (c) HRTEM images, and (d) STEM images along with (e) EDX analysis.

The presence of the CsCuI_3_ perovskite inside PCN-222 is revealed using UV–Vis absorption spectroscopy by the appearance of the characteristic absorption band at 360 nm. Additionally, the composites exhibit different fluorescence spectra than the individual components (Figure 4a,b), indicating that incorporation of CsCu_2_I_3_ inside the porphyrin MOF establishes an electronic guest (perovskite)–host (PCN-222) interaction that alters the emission properties. Particularly notable is the position of λ_em_ for the maximum emission intensity that appears to be blue-shifted with respect to 1D CsCu_2_I_3_, which typically appears at λ_em_ > 500 nm. The λ_em_ value of about 450 nm corresponds better to nanoparticles of Cu perovskites due to their small particle size (see below).

The presence of the CsCu_2_I_3_ perovskite was also evidenced by X-ray photoelectron spectroscopy (XPS) measurements. Figure S7 displays the XPS spectra of the Cu (2p) region for the whole series of CsCu_2_I_3_@PCN-222(Fe). All samples exhibit strong Cu 2p_1/2_ (952.4 eV) and Cu 2p_3/2_ (932.4 eV) peaks without any noteworthy Cu(II) satellites at ∼942 and ∼962 eV, demonstrating that the oxidation state corresponds predominantly to Cu(I) in agreement with the formation of the lead-free CsCu_2_I_3_ perovskite.^29^ Interestingly, these results also indicate that the mesoporous MOF environment successfully prevents the oxidation of Cu^+^ to Cu^2+^ even after prolonged thermal treatments. To further confirm the synergistic effects of the PCN-222(Fe) host and CsCu_2_I_3_ nanocrystals, XPS peaks of Cs, Cu, and I in CsCu_2_I_3_ and CsCu_2_I_3_@PCN-222(Fe) samples were compared (Figure S8). For CsCu_2_I_3_, the characteristic peak of Cs 3d can be deconvoluted in two components: Cs^+^ at 737.6 eV and Cs–I at 723.69 eV. The Cu 2p spectrum peak is fitted to a Cu–I bond at 931.20 eV.^27,30,31^ The I 3d characteristic peaks are attributed to two bonds, Cs–I at 618.11 eV and Cu–I at 619.37 eV.^27,30^ It should be noted that the C 1s and O 1s peaks, likely coming from the solvent, are also detected. The notable binding energy shifts are observed in the XP spectra of the composite compared to those of pristine CsCu_2_I_3_ (see Figure S8 for details). Also, the elemental signals of the CsCu_2_I_3_@PCN-222(Fe) hybrid show relatively broad peaks as compared to those of CsCu_2_I_3_.

Images of the CsCuI_3_ perovskite inside the MOF crystals could be obtained by cutting thin nanometric slices of the CsCu_2_I_3_@PCN-222(Fe) material using fast ion bombardment (FIB) and placing these slices in a high-resolution scanning transmission electron microscope. The specimens were thin enough to allow electron transmission imaging. As can be seen in Figure 4c, although large dark dots of about 100 nm corresponding to the CsCuI_3_ perovskite were already observed at low magnification, they are most probably due to artifacts formed during the FIB process. At higher magnifications, the STEM images show a very good dispersion of CsCuI_3_ dots all around the PCN-222(Fe) matrix (Figure 4d). A considerable number of particles between 2 and 4 nm were clearly visualized in the STEM images, accompanied by larger particles between 5 and 10 nm. It is proposed that the larger particles are formed by agglomeration of the smaller CsCuI_3_ particles as a consequence of the FIB manipulation. Dark-field images and EDX analysis confirm that these dots are constituted by Cu, Cs, and I (Figure 4e). Therefore, the HR-STEM study shows a very good dispersion of CsCu_2_I_3_ with a considerable fraction of CsCuI_3_ particles having a size commensurate with PCN-222 channel dimensions of about 3.2 nm, compatible with the internal incorporation of CsCu_2_I_3_.

### 3.2 Catalytic Properties of PCN-222(Fe)-Encapsulated CsCu_2_I_3_

Copper(I)-catalyzed intermolecular alkyne-azide cycloaddition (CuAAC) reaction, also named as the Sharpless “click” reaction, was reported in 2001^50^ and used for the synthesis of triazoles as fine chemicals^32^ and for the efficient covalent surface functionalization and covalent attachment under mild conditions.^21^ Therefore, the catalytic activity of the CsCu_2_I_3_@PCN-222(Fe) composites was first evaluated for the CuAAC reaction. In this regard, the catalytic performance of the CsCu_2_I_3_@PCN-222(Fe) hybrids was investigated in a new click reaction version, one-pot three-component type. The results are summarized in Table 1. Remarkably, CsCu_2_I_3_@PCN-222(Fe) (15 min-r.t., with 0.8 wt % copper and 11.47 wt % zirconium) promoted the reaction at ambient temperature in aqueous media, a naturally abundant and green solvent, and gave the isolated 1,4-diphenyltriazole product with a yield of 98% and a high selectivity of 99.9% after 85 min (Table 1, entry 1). The reaction is stereoselective with the exclusive formation of the 1,4-isomer without any observation of the 1,5-isomer.^33^ Water and water-miscible organic solvent mixtures have been reported as CuAAC reaction media.^32,34−36^ Organic reactions in water lacking an organic solvent offer a significant advantage from the environmental point of view, provided that substrates and reagents are soluble in this medium. Since the pore surface is predominantly lined with the hydrophobic organic linkers, the PCN framework has a preference in aqueous solution for adsorption of the organic starting materials into the cavity *via* “hydrophobic–hydrophobic interactions”,^36^ thereby increasing the concentration of substrates near the active sites, which increases the catalytic reaction rate and selectivity. When the Cu content increased to 1.93 wt % (CsCu_2_I_3_@PCN-222(Fe)-15 min-60 °C, 13.66 wt % zirconium), although the BET surface area and porosity slightly decreased, the activity was almost coincident with that of CsCu_2_I_3_@PCN-222(Fe)-15 min-r.t. (yield of 98% at 80 min) (Table 1, entry 2). When CsCu_2_I_3_ was increased to 11.85 wt % copper (2.94 wt % zirconium) in the case of CsCu_2_I_3_@PCN-222(Fe)-3.5 h-60 °C, the product yield of 98% was achieved at a shorter reaction time of 30 min in comparison with the 15 min samples (Table 1, entry 3). The activity of CsCu_2_I_3_@PCN-222(Fe) with 13.88 wt % copper (CsCu_2_I_3_@PCN-222(Fe)-3.5 h-r.t.) was almost the same as that of CsCu_2_I_3_@PCN-222(Fe)-3.5 h-60 °C with similar porosity and metal content (Table 1, entry 4). The data revealed that a balance between porosity and CsCu_2_I_3_ loading seems to play an essential role in the performance enhancement of CsCu_2_I_3_@PCN-222(Fe) in this reaction. In other control tests, the catalytic activity of the constructed PCN-222(Fe) and CsCu_2_I_3_ was also considered for comparison (Table 1, entries 5–7). PCN-222(Fe) showed no catalytic activity and gave no product after 1 h (Table 1, entry 5). For CsCu_2_I_3_, the obtained yield was lower than that of the porous CsCu_2_I_3_@PCN-222(Fe)-3.5 h (Table 1, entry 6 *vs* entries 3 and 4). When physically mixed PCN-222(Fe) and CsCu_2_I_3_ were used as a catalyst, the product yield was still lower than that of CsCu_2_I_3_@PCN-222(Fe)-3.5 h-60 °C (Table 1, entry 7).

**Table 1:**
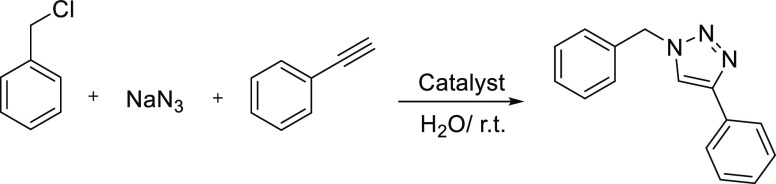
One-Pot Three-Component CuAAC Reaction by Different Catalysts^a^

entry	catalyst (mg)	time (min)	yield (%)^b^
1	CsCu_2_I_3_@PCN-222(Fe)-15 min-r.t. (10)	85	98
2	CsCu_2_I_3_@PCN-222(Fe)-15 min-60 °C (10)	80	98
3	CsCu_2_I_3_@PCN-222(Fe)-3.5 h-60 °C (10)	30	98
4	CsCu_2_I_3_@PCN-222(Fe)-3.5 h-r.t. (10)	∼30	98
5	PCN-222(Fe) (7)	60	
6	CsCu_2_I_3_ (7)	30	80
7	PCN-222(Fe) (7) + CsCu_2_I_3_ (7)	30	85

a Reaction conditions: benzyl chloride (0.5 mmol), NaN_3_ (1 mmol), phenylacetylene (1 mmol), water (2 mL), and catalyst.

b Purified yield.

To show the excellent catalytic performance of our host–guest material, the activity of CsCu_2_I_3_@PCN-222(Fe) was compared with that of previously reported catalysts in this reaction, as given in Table S3. As can be seen in Table S3, the previously reported catalysts such as CuI@UiO-67-IM (IM = imidazolium salt),^35^ CuSO_4_·5H_2_O/sodium ascorbate/β-cyclodextrin,^36^ Cu-tetracatechol metallopolymer,^37^ CuI 1D polymeric coordination complex,^38^ and self-assembled poly(imidazole-acrylamide)-Cu/sodium ascorbate^39^ generally exhibit lower catalytic activity or low selectivity and require higher temperature or longer reaction time in comparison with the use of the CsCu_2_I_3_@PCN-222(Fe) catalyst. Moreover, CsCu_2_I_3_@PCN-222(Fe) was successfully reused and maintained its outstanding performance for the click reaction in terms of both activity and selectivity (Figure S9). The superior performance of CsCu_2_I_3_@PCN-222(Fe) is attributed to the stabilization by confinement and isolation of the CsCu_2_I_3_ active sites within the MOF while still being accessible due to the porosity of the MOF host.

Tandem/cascade reactions, consisting in multistep sequential reactions performed in one pot, are exciting reactions in modern chemistry that can minimize wastes, diminish energy consumption, avoid intermediate workups, and optimize the use of resources.^40^ We then were interested in investigating the activity of the synthesized CsCu_2_I_3_@PCN-222(Fe) composites as heterogeneous catalysts for one-pot cascade selective photo-oxidation of benzyl alcohols/*in situ* Knoevenagel condensation with malononitrile under visible LED illumination using oxygen (balloon, 1 atm.) as a green oxidant in acetonitrile (Table 2). There was nearly no product in the absence of either CsCu_2_I_3_@PCN-222(Fe)-3.5 h-60 °C or visible light (Table 2, entries 1 and 2). In the presence of CsCu_2_I_3_@PCN-222(Fe)-3.5 h-60 °C, the reaction proceeded efficiently upon LED irradiation. The isolated yield of the benzylidenemalononitrile product reached 96% after 15 h with full conversion of benzyl alcohol (>99%) (Table 2, entry 3). It was also found that CsCu_2_I_3_-loaded PCN-222(Fe) with the lowest amount of copper (CsCu_2_I_3_@PCN-222(Fe)-15 min-60 °C) could also promote the reaction and afforded a product yield of 67% within 15 h (Table 2, entry 4). When N_2_ was used instead of O_2_, the product yield was suppressed significantly (Table 2, entry 5), showing the role of O_2_ as an oxidant. In a control experiment with pure CsCu_2_I_3_ under identical conditions, the yield was very low even after a prolonged time period (Table 2, entry 6). In additional experiments using pristine PCN-222(Fe) MOF and its physical mixture with CsCu_2_I_3_, the product yields were still lower than that of CsCu_2_I_3_@PCN-222(Fe)-3.5 h-60 °C (Table 2, entries 7 and 8). These results indicate that the good performance of CsCu_2_I_3_@PCN-222(Fe) in this reaction is ascribed to the existence of the mesoporous MOF host and CsCu_2_I_3_ components, establishing a synergistic effect between both of them.

**Table 2:**
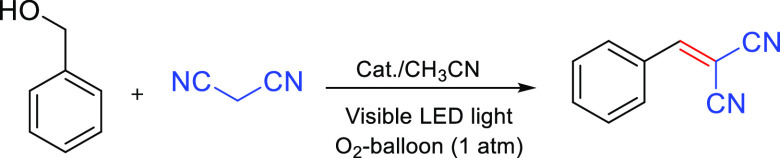
Benzylidenemalononitrile Formation *via* One-Pot Selective Photo-oxidation/Knoevenagel Condensation Cascade Reaction under Different Conditions^a^

entry	catalyst (mg)	time (h)	yield (%)^b^
1^c^		15	
2^d^	CsCu_2_I_3_@PCN-222(Fe)-3.5 h-60 °C (15)	15	trace
3	CsCu_2_I_3_@PCN-222(Fe)-3.5 h-60 °C (15)	15	96
4	CsCu_2_I_3_@PCN-222(Fe)-15 min-60 °C (15)	15	67
5^e^	CsCu_2_I_3_@PCN-222(Fe)-3.5 h-60 °C (15)	15	
6	CsCu_2_I_3_ (11)	24	34
7	PCN-222(Fe) (10)	24	60
8	PCN-222(Fe) (10) + CsCu_2_I_3_ (11)	15	71

a Reaction conditions: benzyl alcohol (0.5 mmol), malononitrile (0.75 mmol), acetonitrile (2–3 mL), and catalyst.

b Purified product.

c In the absence of the catalyst.

d In the absence of visible LED light irradiation.

e N_2_ instead O_2_.

The scope of the CsCu_2_I_3_@PCN-222(Fe) photocatalyst was then surveyed for electron-donating and electron-withdrawing groups, *e.g.*, methyl and chloride, on benzyl alcohols, as shown in Table 3. Under typical conditions, the aromatic alcohols were effectively converted to their corresponding benzylidenemalononitrile products in good yields with high selectivity (>99%) (Table 3), indicating the potential utility of this photocatalyst.

**Table 3:**
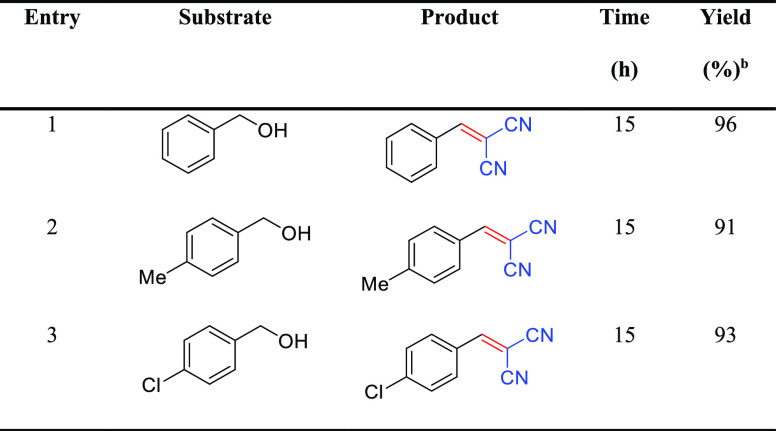
CsCu_2_I_3_@PCN-222(Fe)-Catalyzed Selective One-Pot Cascade Synthesis of Benzylidenemalononitriles from Benzyl Alcohols^a^

a Reaction conditions: benzyl alcohol (0.5 mmol), malononitrile (0.75 mmol), acetonitrile (2–3 mL), and catalyst (15 mg).

b Purified products.

Table S4 also shows the outstanding performance of CsCu_2_I_3_@PCN-222(Fe) compared with the other reported (photo)catalysts^41−45^ for this reaction. Several types of photocatalytic/catalytic systems including NH_2_-MIL-101(Fe),^42^ g-C_3_N_4_/FeWO_4_,^41^ Au(III)@Cu(II)-MOF,^43^ Cu(II)/amine bifunctional MOF,^44^ and Zr-MOF-NH_2_^45^ have been reported for the production of benzylidenemalononitrile. However, the procedures reported so far have several disadvantages including poor stability, the presence of noble metals, long reaction, the use of oxidant (*tert*-butyl hydroperoxide or hydrogen peroxide), artificial UV light, and an excess of malononitrile as well as high temperatures and high catalyst loadings, which diminish the interest of the process. On the other hand, in the recent years, due to global warming and the shift in the energy sources, conventional synthetic techniques have moved toward the use of visible light photocatalytic systems, employing LEDs that significantly reduce energy consumption.^46^ Notably, the multifunctional CsCu_2_I_3_@PCN-222(Fe) worked well without any additive, at low catalyst loading, and with a low power light source.

Two of the main advantages of using a heterogeneous (photo)catalyst are recyclability and reusability. Therefore, the reuse experiments were performed under the best reaction conditions as presented in Figure S10. The results presented that the hybrid CsCu_2_I_3_@PCN-222(Fe) photocatalyst could be reused for at least four sequential reaction cycles without substantial decay in efficiency (Figure S10) after its recovery by centrifugation and washing. Atomic absorption spectroscopy (AAS) analysis of the supernatant revealed insignificant copper (0.9% of the total Cu amount in the photocatalyst) and iron leaching (0.4%). The comparison of fresh and reused PXRD profiles (Figures S11) and SEM images (Figure S12*versus*Figure 2a) revealed that CsCu_2_I_3_@PCN-222(Fe) has retained its crystallinity and particle morphology after the cascade reaction. In addition, EDX elemental analysis of the photocatalyst indicated that the metal contents do not change significantly after its reuse (Figures S13 and S14*versus*Tables S1 and S2). The recovered catalyst was further assessed by using TGA, which shows the same profile as that of the fresh one (Figure S15). All the available data indicate that CsCu_2_I_3_@PCN-222(Fe) is an active, stable, and reusable multifunctional catalyst. These stability data are remarkable considering the small size of the particles and the instability of iodide perovskites.

The work showed that the integration of a semiconductor-like porphyrinic MOF, PCN-222(Fe), with perovskite CsCu_2_I_3_ nanocrystals results in synergistic effects derived from confinement and affords an optimized and efficient catalytic system for the tandem reaction.

### 3.3 Mechanistic Studies

**Scheme 1:**
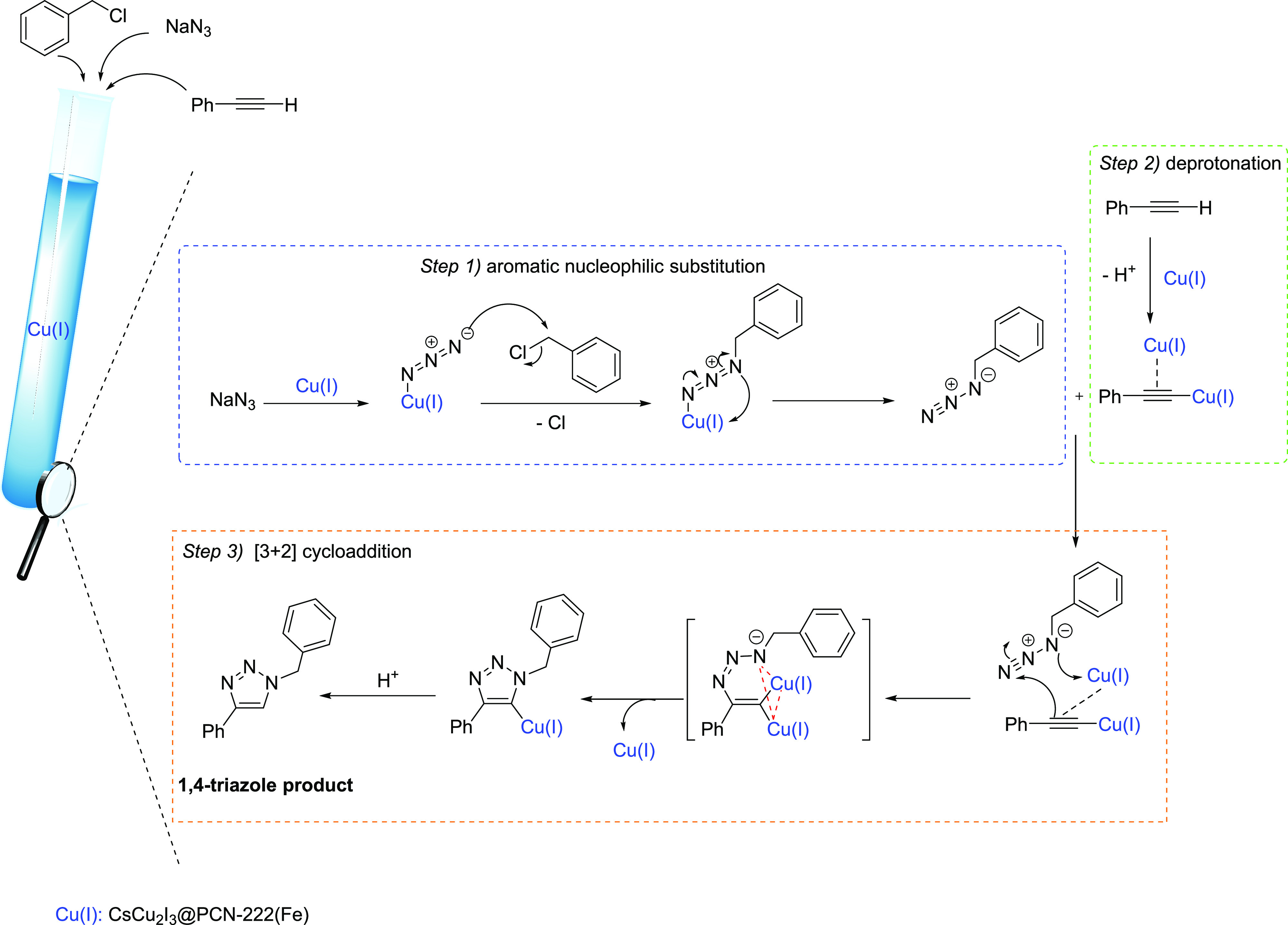
Proposed Mechanism for the Click Synthesis of 1,4-Diphenyl-1,2,3-triazole by Using the CsCu_2_I_3_@PCN-222(Fe) Catalyst

A plausible mechanism for the three-component azide-alkyne 1,3-dipolar cycloaddition reaction toward the regioselective synthesis of 1,4-diphenyl-1,2,3-triazole is depicted in Scheme 1. CsCu_2_I_3_@PCN-222(Fe) including well-dispersed Cu(I) species^32^ composing CsCu_2_I_3_ (Figures S7 and S8) can act as a heterogeneous multifunctional catalyst promoting the various sequential steps: the first step is the *in situ* generation of 1,4-phenyl azide *via* aromatic nucleophilic substitution reaction between sodium azide and benzyl bromide, the second step is the deprotonation of the terminal hydrogen of phenyl acetylene, and the third step is the [3 + 2] cycloaddition reaction between the deprotonated phenyl acetylene and the *in situ*-formed azide to afford the desired 1,4-isomer product as shown in Scheme 1. It should be noted that the mesoporous MOF host allows the accessibility of these reactants to the Cu(I) sites and prompts the reactions within the cavities.

To gain key insights into the mechanistic scenarios of the one-pot cascade transformation over CsCu_2_I_3_@PCN-222(Fe), additional control experiments were carried out (see Scheme S1 and also the experiments given in Table 2). In the cascade reaction between benzyl alcohol and malononitrile, benzaldehyde appears as an intermediate. Photocatalytic oxidation of benzyl alcohol to benzaldehyde takes place without over-oxidation to benzoic acid, which was barely detected during the course of the reaction (Scheme S1a). In another control test, under the same condition, when benzyl alcohol was replaced by benzaldehyde, the desired benzylidenemalononitrile was produced quantitatively after a short reaction time of ∼30 min even in the dark (Scheme S1b,c). These tests confirmed that the condensation reaction between benzaldehyde and malononitrile is easier and faster (Scheme S1) than the photo-oxidation process, which is the rate-determining step in the cascade reaction. The controls reveal that the host–guest hybrid material serves as a multifunctional (photo)catalyst including also both Lewis acidic sites such as Fe^3+^, Zr^+4^, Cu^+^, and Cs^+^ (see Scheme S1 for additional experiments and Figure S16) and redox-active sites of PCN-222(Fe) and lead-free CsCu_2_I_3_ perovskite as the light absorber. Both components operate synergistically.

**Figure 5:**
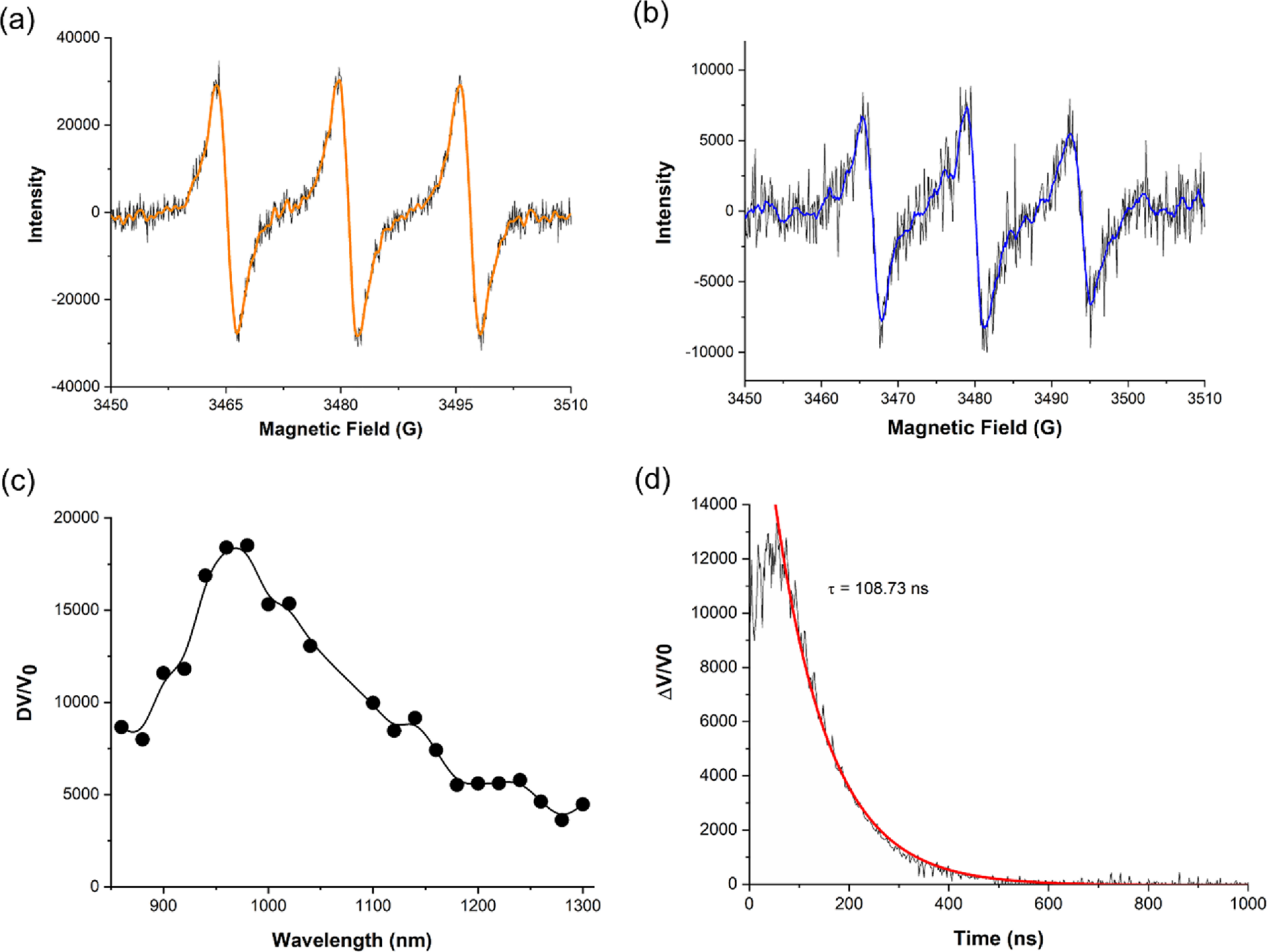
EPR spectra of samples of (a) TEMP or (b) DMPO with CsCu_2_I_3_@PCN-222(Fe) and O_2_ under visible light irradiation, (c) time-resolved spectrum corresponding to ^1^O_2_ photoluminescence, and (d) temporal signal decay profile of ^1^O_2_ phosphorescence recorded for CsCu_2_I_3_@PCN-222(Fe) upon 355 nm laser excitation.

To further identify the prevalent reactive oxygen species (ROS) in the present system promoting the photo-oxidation step, inhibition experiments by adding quenchers such as 1,4-benzoquinone (superoxide radical anion (O_2_^•–^) scavenger), sodium azide (singlet oxygen (^1^O_2_) scavenger), and methanol (hole (h^+^) scavenger) to the reaction mixture were performed (Figure S17). The quenching study is compatible with the *in situ* formation of O_2_^•–^ and ^1^O_2_ (main species) as well as h^+^ (minor species) during the photocatalytic process, as assessed by the decrease in product yield. Electron paramagnetic resonance (EPR) measurements were then performed to quantify the *in situ*-generated O_2_^•–^ and ^1^O_2_ as the ROS in this system (Figure 5). Using 2,2,6,6-tetramethylpiperidine (TEMP) as a specific ^1^O_2_ trapping agent, a strong triplet signal of an intensity ratio of 1:1:1, corresponding to TEMPO, was detected after light irradiation, which confirmed the formation of ^1^O_2_ (Figure 5a). To confirm O_2_^•–^ generation, 5,5-dimethyl-1-pyrroline *N*-oxide (DMPO), as a specific O_2_^•–^ trapping agent, was used, which generated multiline EPR signals after light irradiation (Figure 5b), corresponding to DMPO-O_2_^•–^ and nitroxide-related radicals^47^ resulted from ^1^O_2_/O_2_^•–^-driven DMPO decomposition. It should be noted that no radical and no EPR signal were detected under dark conditions.

Furthermore, the lifetime of the singlet oxygen formed within the porous CsCu_2_I_3_@PCN-222(Fe) photocatalyst was measured by the time-resolved phosphorescence technique.^48^ The method permits one to quantify directly the generation of ^1^O_2_ by detecting its characteristic phosphorescence in the near-infrared (NIR) region (Figure 5c,d). Upon single-pulse laser excitation at 355 nm, the time-resolved NIR phosphorescence showed a ^1^O_2_ lifetime of up to 100 ns for the CsCu_2_I_3_@PCN-222(Fe) photocatalyst.

The above-described arguments evidently confirm that CsCu_2_I_3_@PCN-222(Fe) is cooperatively capable of generating ^1^O_2_, *via* an energy transfer pathway, and O_2_^•–^, *via* a charge transfer (CT)/single electron transfer (SET) pathway, during the process while acting as an Lewis acid catalyst.

**Scheme 2:**
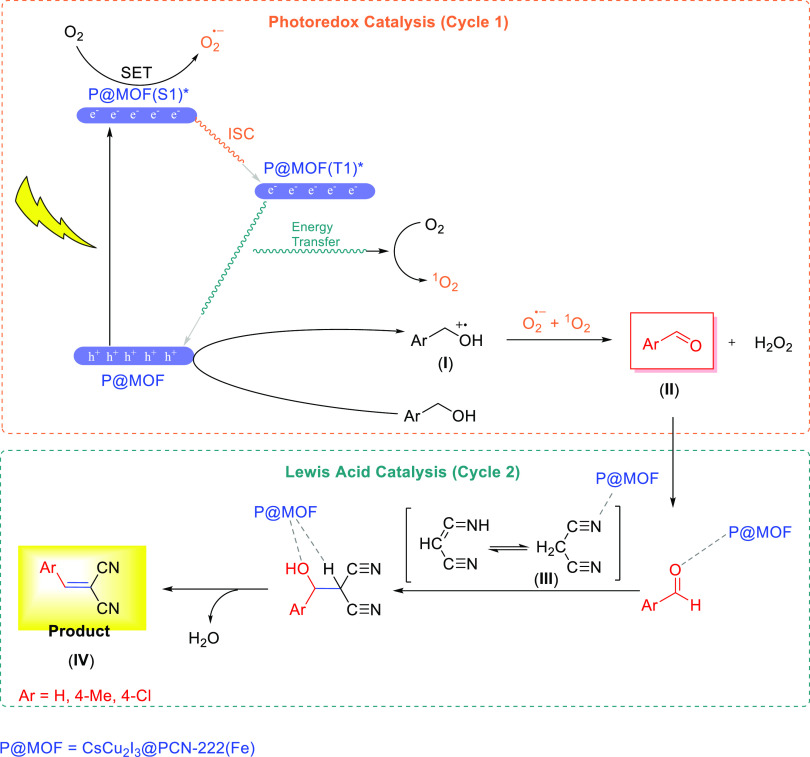
Proposed Mechanism for the Condensation Cascade Transformation over CsCu_2_I_3_@PCN-222(Fe)

Hence, according to the control experiments, quenching and EPR tests, XPS data, time-resolved phosphorescence, and literature survey,^22,49^ a possible mechanism for the one-pot selective photo-oxidation/Knoevenagel condensation cascade reaction can be proposed. The mechanism is depicted in Scheme 2. Upon light irradiation, the CsCu_2_I_3_@PCN-222(Fe) multifunctional catalyst (here simply denoted as P@MOF) is first excited to form the singlet excited state of the P@MOF, [P@MOF(S_1_)]*. [P@MOF(S_1_)]* is oxidized by adsorbed O_2_ to form O_2_^•–^ over a single electron transfer (SET) process. Alternatively, [P@MOF(S_1_)]* can relax vibrationally to a lower energy triplet state, [P@MOF(T_1_)]*, through an intersystem crossing (ISC) process and can be quenched further by O_2_ to generate ^1^O_2_ over an energy transfer. The generated hole (h^+^) rapidly oxidizes the benzyl alcohol to generate radical cation species (**I**) as the intermediate, which can consequently react with the photoformed ROS (O_2_^•–^ and ^1^O_2_) to produce the corresponding aldehyde product (**II**) and H_2_O_2_ by-product. The observed selectivity of the P@MOF is likely regulated by the micro/meso-hierarchical hydrophobic structure, which allows the free diffusion of substrates/products and prevents over-oxidation by the same species of the hybrid catalyst. Next, the P@MOF including the Lewis acid sites (such as Fe^3+^, Zr^+4^, Cu^+^, and Cs^+^) activates the *in situ*-produced aldehyde and malononitrile (**III**) for a nucleophilic addition of the Knoevenagel coupling reaction toward the formation of the final product (**IV**). Subsequently, the (photo)catalyst is ready for another round of tandem reaction.

## 4 Conclusions

Briefly, tetrametallic multifunctional porous CsCu_2_I_3_@PCN-222(Fe) hybrid materials have been creatively planned and fabricated by post-synthetic modification of iron-porphyrin PCN-222(Fe) with the earth-abundant and lead-free CsCu_2_I_3_ perovskite *via* the antisolvent protocol using the MOF and the same amount of CsCu_2_I_3_ precursors (CuI and CsI). Characterization of CsCu_2_I_3_@PCN-222(Fe) rods shows unique structural and functional properties arising from the collective active sites of the CsCu_2_I_3_ nanoparticles incorporated inside the MOF pores, providing stabilization of the iodide perovskite. These properties have allowed the use of CsCu_2_I_3_@PCN-222(Fe) as a heterogeneous catalyst, revealing a remarkable catalytic activity for the three-component click reaction and one-pot selective photo-oxidation/Knoevenagel domino reaction with oxygen (1 atm) under visible light. The CsCu_2_I_3_@PCN-222(Fe) composite performs better than benchmark catalysts for these two processes. The activity appears to be a balance between porosity and CsCu_2_I_3_ loading. Outstandingly, CsCu_2_I_3_@PCN-222(Fe) was stable under reaction conditions and can be reused several times. Considering the diversity of halide perovskites, our results open the way to exploit the potential in the catalysis of these materials upon encapsulation inside MOFs.

## Supporting Information Available

Details of characterizations (SEM images, EDS analyses, mapping elemental analyses, N_2_ adsorption–desorption measurements, pore size distributions, UV–Vis DRS spectra, Tauc plots, TGA profiles, XPS spectra, PXRD patterns, melting points, and NMR spectra), control experiments, recycling tests, inhibition investigation, and comparison tables with other materials (PDF)
