# Supplementary material for: CsCu2I3 Nanoparticles Incorporated within a Mesoporous Metal–Organic Porphyrin Framework as a Catalyst for One-Pot Click Cycloaddition and Oxidation/Knoevenagel Tandem Reaction
Source: ACS Appl Mater Interfaces. 2022 Aug 8;14(32):36515–26. doi: 10.1021/acsami.2c04364 (PMC9940116; doi:10.1021/acsami.2c04364)
Supplement: Supplementary file 1 — am2c04364_si_001.pdf [file am2c04364_si_001.pdf]

Supporting Information

$CsCu_2I_3$ nanoparticles incorporated within a mesoporous metal-organic porphyrin framework as a catalyst for one-pot click cycloaddition and oxidation/Knoevenagel tandem reaction

Saba Daliran,[a] Mostafa Khajeh,[a,*] Ali Reza Oveisi,[a,*] Josep Albero,[b] and Hermenegildo García[b,*]

[a] Department of Chemistry, University of Zabol, P.O. Box: 98615-538, Zabol, Iran

[b] Departamento de Química and Instituto de Tecnología Química CSIC-UPV, Universitat Politècnica de València, Av. de los Naranjos s/n, 46022 Valencia, Spain

* Corresponding authors.

E-mail addresses: m_khajeh@uoz.ac.ir (M. Khajeh), aroveisi@uoz.ac.ir & alir.oveisi@gmail.com (A. R. Oveisi), hgarcia@qim.upv.es (H. García)

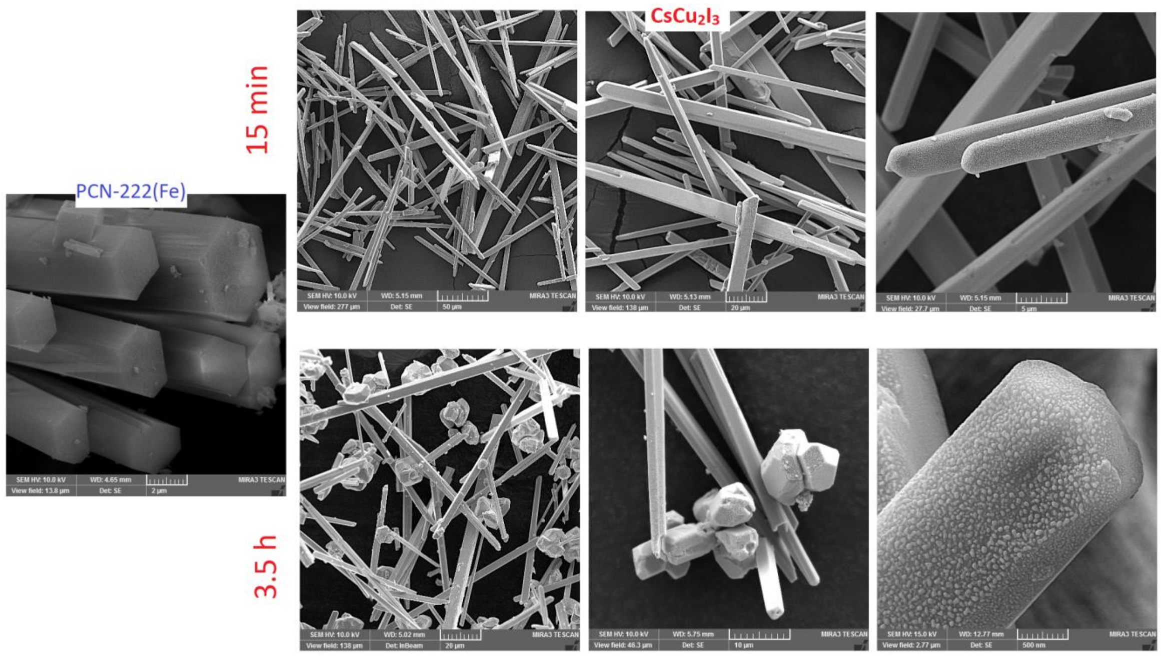

Figure S1. SEM images of individual $CsCu_2I_3$ and pristine PCN-222(Fe) MOF.

Table S1. EDX analyses of the $CsCu_2I_3$@PCN-222(Fe) samples obtained in different synthesis time and temperature (15 min-r.t., 3.5 h-r.t., 15 min-60 °C, and 3.5 h-60 °C).

Condition	EDX patterns	EDX elemental analysis
**15 min, r.t.**	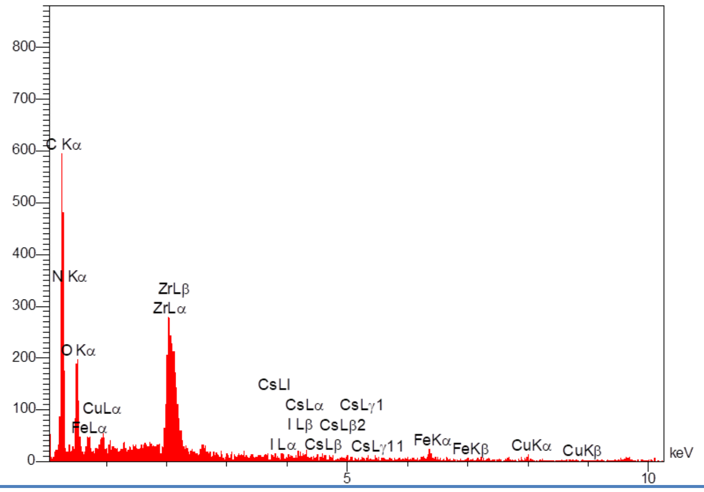	see below

Elt	W%	A%
C	48.99	61.59
N	11.79	12.71
O	24.56	23.18
Fe	0.97	0.26
Cu	0.80	0.19
Zr	11.47	1.90
I	0.49	0.06
Cs	0.93	0.11
	100.00	100.00

3.5 h, r.t.

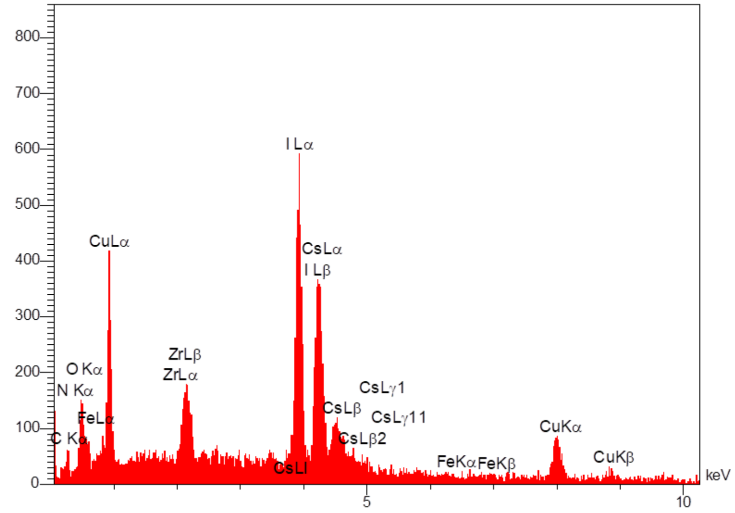

Elt	W%	A%
C	2.97	15.73
N	2.28	10.37
O	5.48	21.81
Fe	0.18	0.18
Cu	13.88	13.90
Zr	2.82	1.97
I	57.91	29.04
Cs	14.60	6.99
	100.00	100.00

15 min, 60 °C

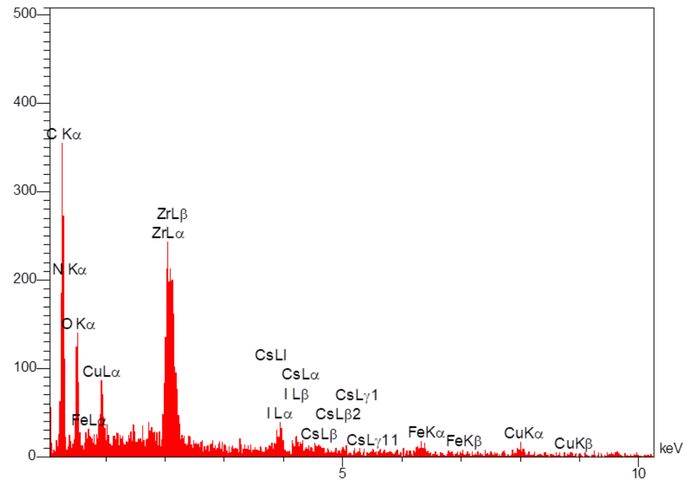

Elt	W%	A%
C	46.02	61.90
N	10.51	12.12
O	21.86	22.09
Fe	1.38	0.40
Cu	1.93	0.49
Zr	13.66	2.42
I	2.83	0.36
Cs	1.81	0.21
	100.00	100.00

3.5 h, 60 °C

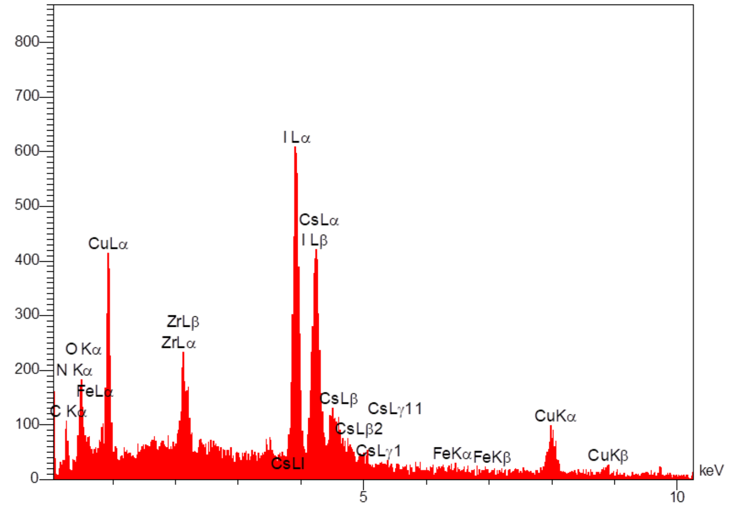

Elt	W%	A%
C	5.91	26.27
N	3.31	12.66
O	5.99	20.03
Fe	0.23	0.22
Cu	11.85	9.96
Zr	2.94	1.72
I	56.78	23.91
Cs	12.99	5.22
	100.00	100.00

Table S2. EDX elemental distribution mapping of the $CsCu_2I_3$@PCN-222(Fe) samples (15 min-r.t., 3.5 h-r.t., 15 min-60 °C, and 3.5 h-60 °C).

Sample	Elemental mapping
15 min, r.t.	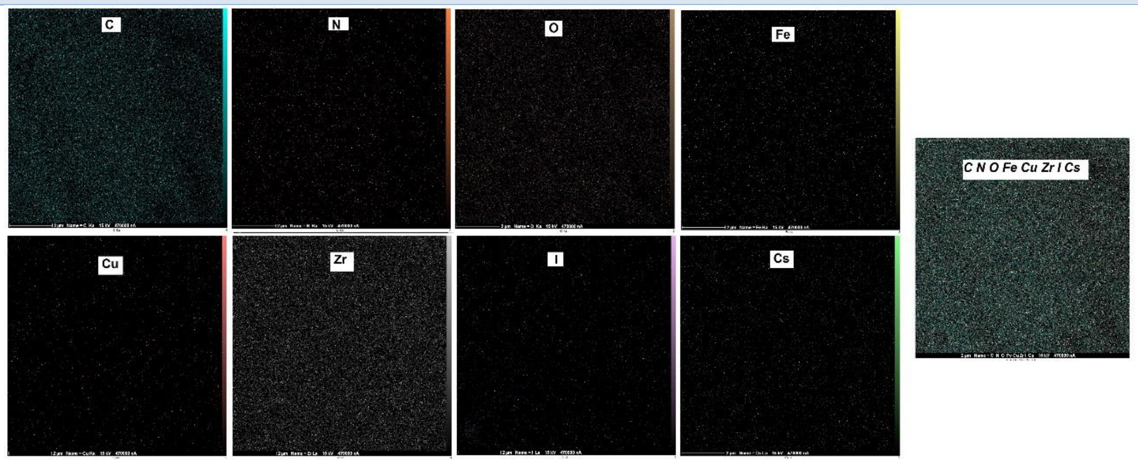

3.5 h, r.t.	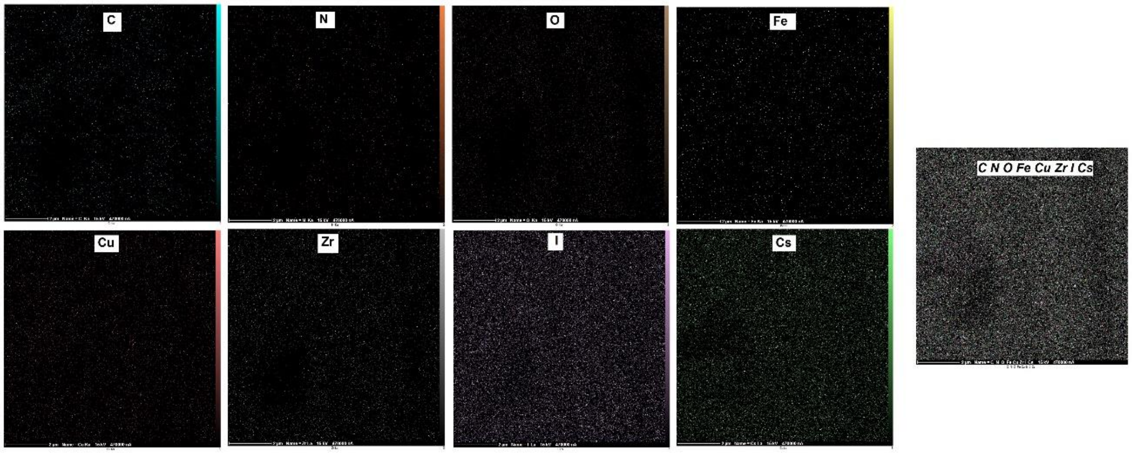
15 min, 60 °C	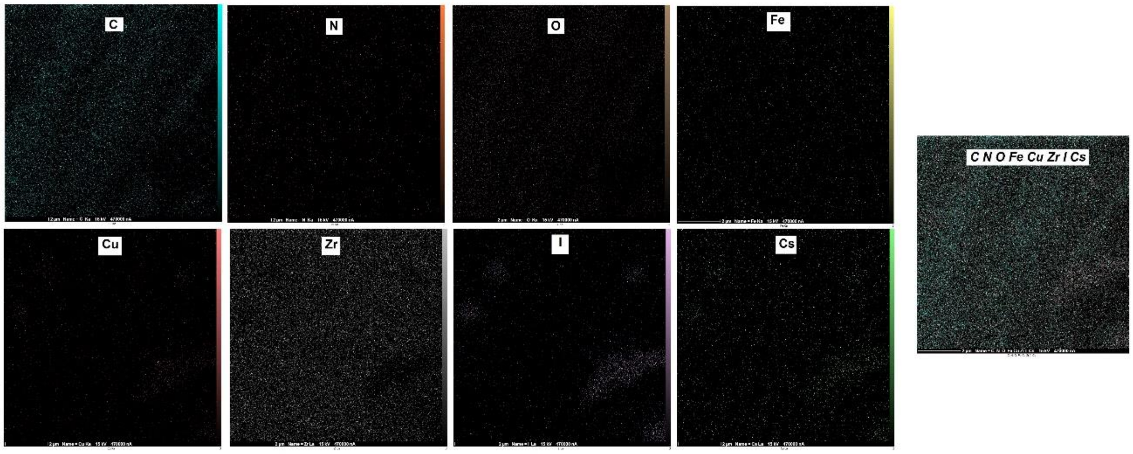
3.5 h, 60 °C	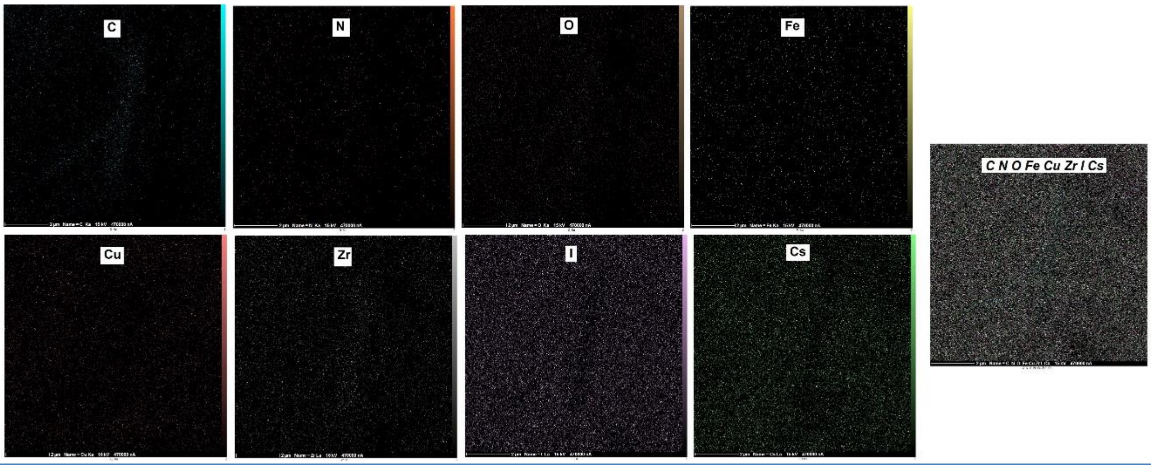

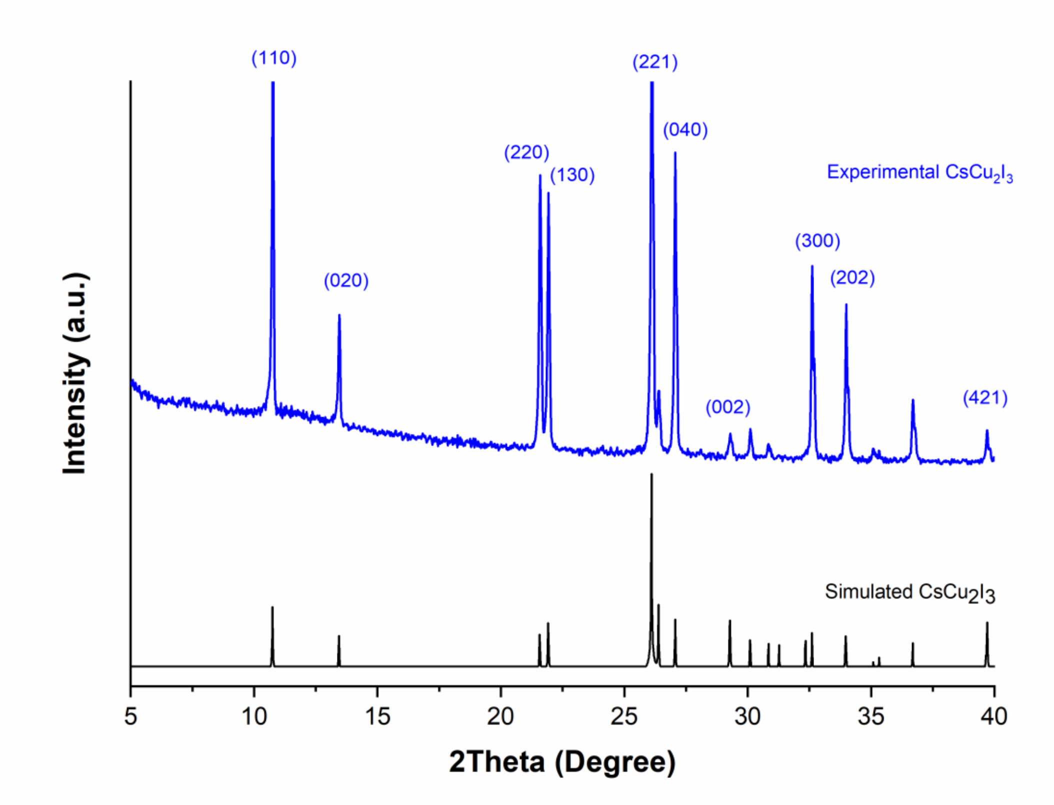

Figure S2. XRD patterns of as-synthesized and the standard data of $CsCu_2I_3$ (PDF #45-0076).

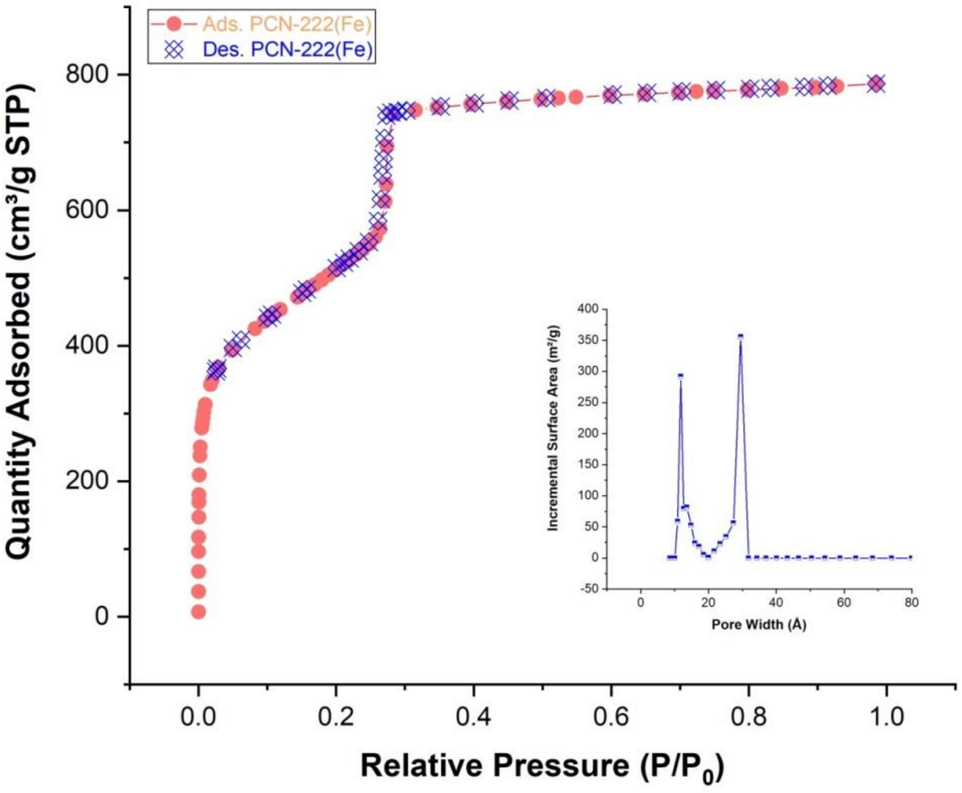

Figure S3. N_2 adsorption-desorption isotherms for PCN-222(Fe) at 77 K. Inset displays its DFT pore size distribution (PSD).

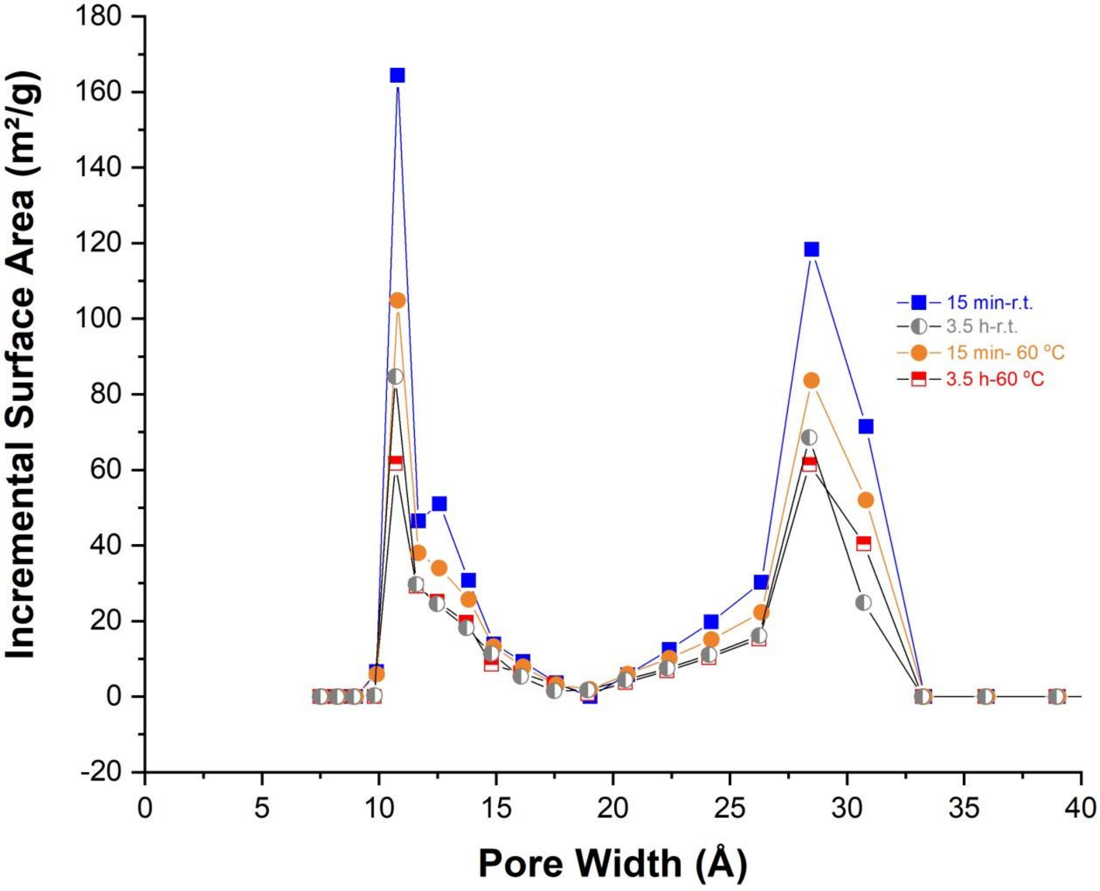

Figure S4. DFT pore size distributions (PSDs) for $CsCu_2I_3$@PCN-222(Fe) hybrid materials spending data measured with N_2 at 77 K.

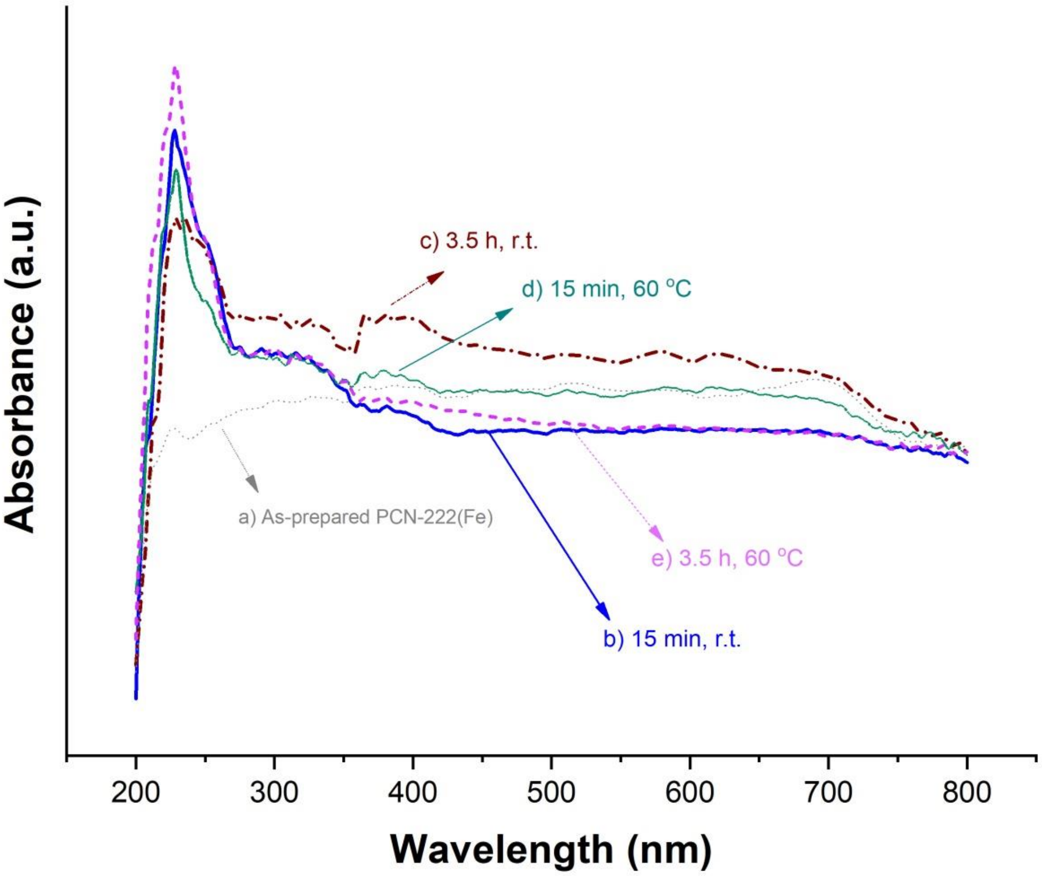

Figure S5. Diffuse reflectance UV/Vis spectra of as-synthesized PCN-222(Fe) and $CsCu_2I_3$@PCN-222(Fe) hybrid materials.

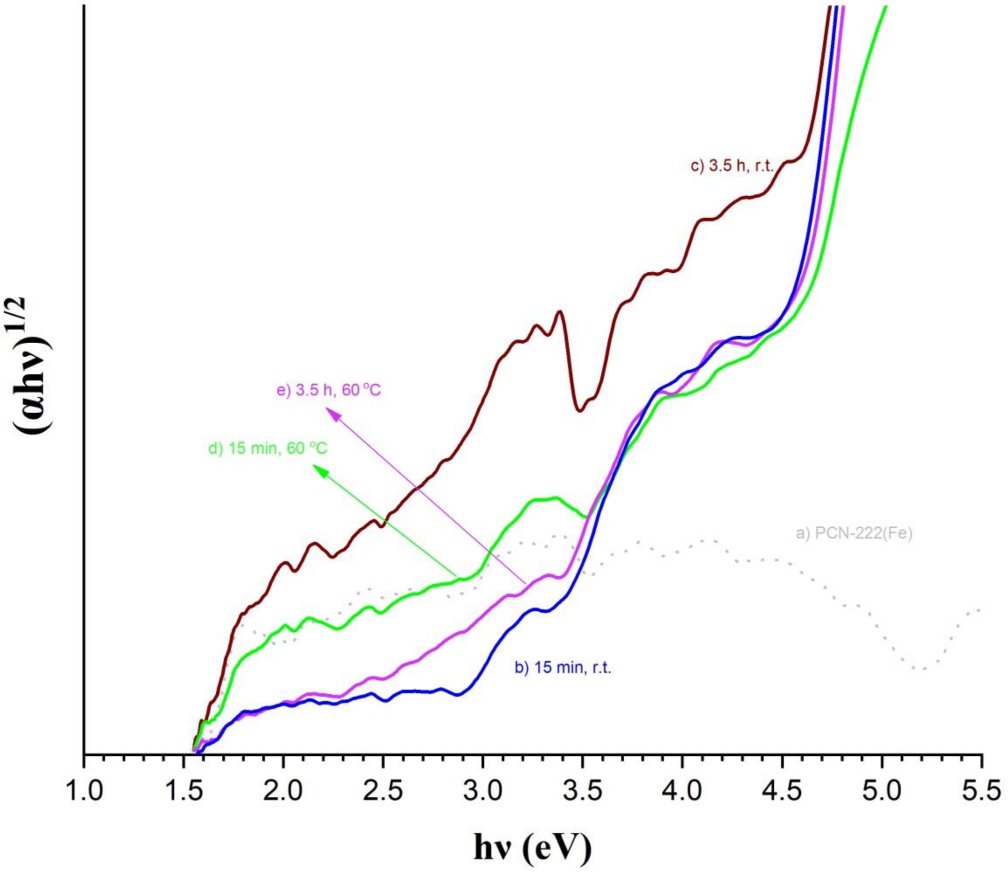

Figure S6. Tauc plots of PCN-222(Fe) and four $CsCu_2I_3$@PCN-222(Fe) hybrid materials.

a)

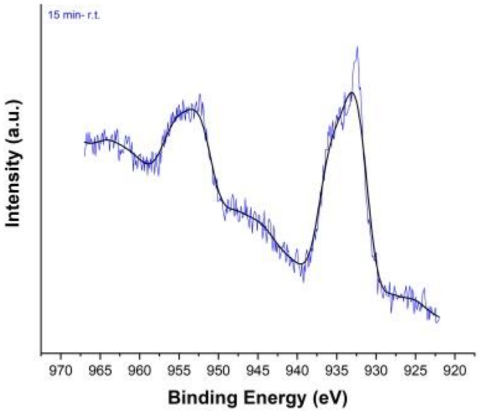

b)

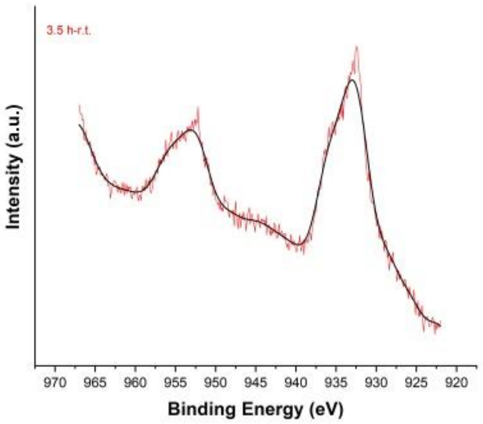

c)

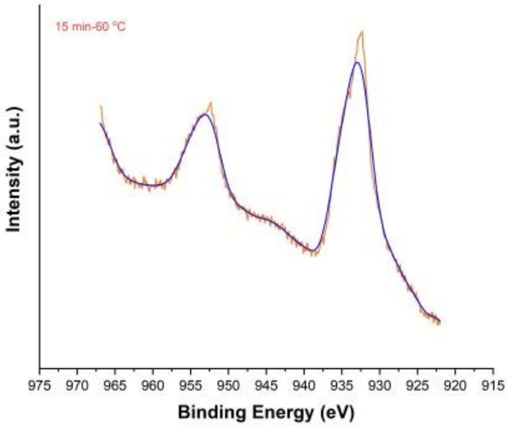

d)

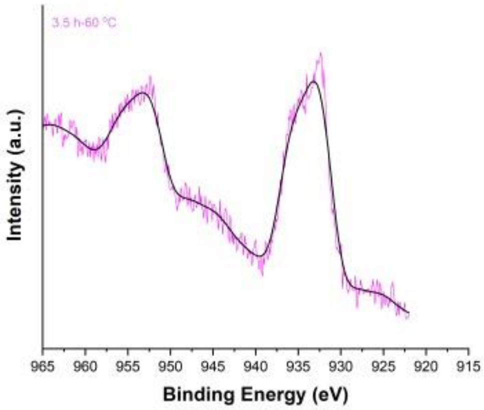

Figure S7. XPS spectra for Cu 2p spectral lines of the four $CsCu_2I_3$@PCN-222(Fe) hybrid materials.

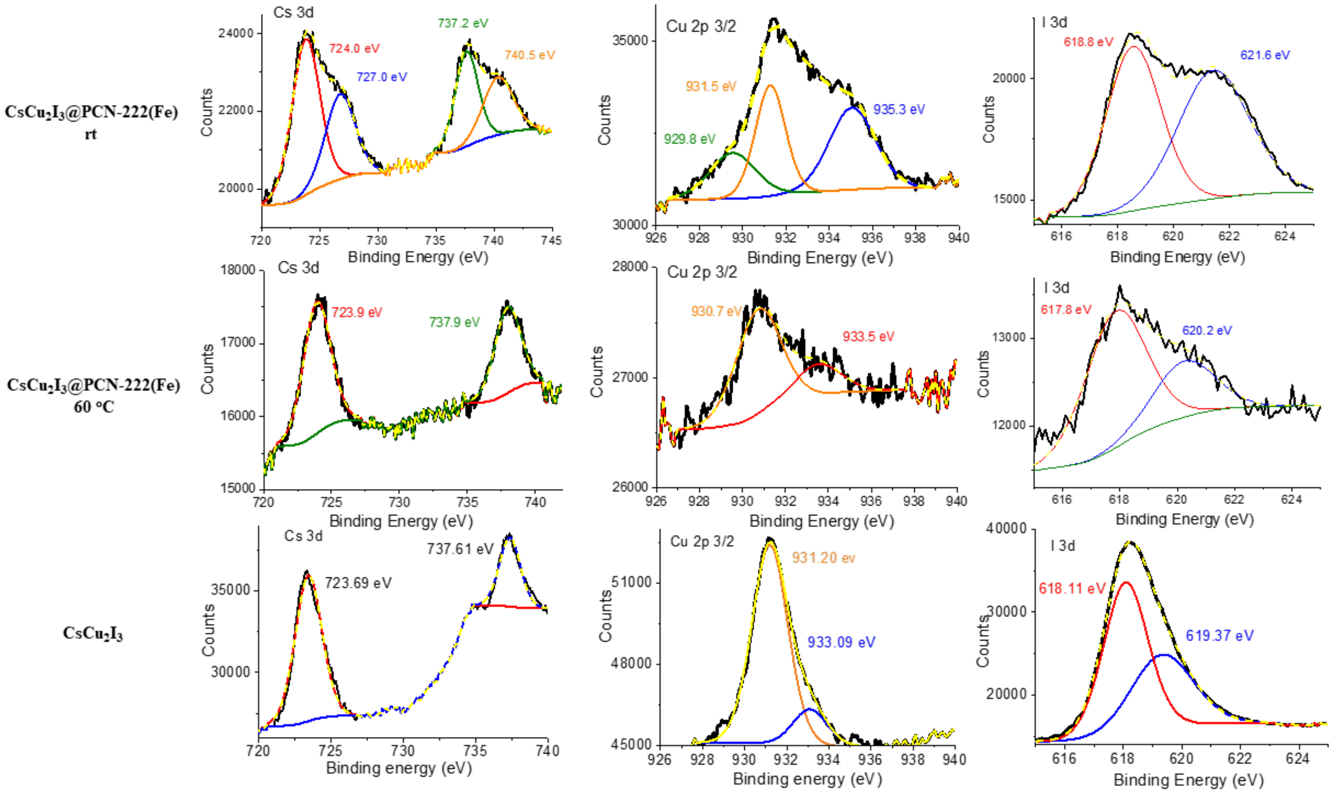

Figure S8. High-resolution XPS Cs 3d, Cu 2p and I 3d spectra of as-synthesized $CsCu_2I_3$@PCN-222(Fe) samples and $CsCu_2I_3$. The difference in the peak binding energy between $CsCu_2I_3$ and, particularly, $CsCu_2I_3$@PCN-222(Fe) 60 °C reflects the interaction of encapsulated $CsCu_2I_3$ with PCN-222(Fe) as host. The 935.3 eV component in Cu 2p present in $CsCu_2I_3$@PCN-222(Fe) can be attributed to Cu^+.

Table S3. Comparison of three-component CuAAC reaction catalyzed by $CsCu_2I_3$@PCN-222(Fe) and the other reported catalysts.

Entry	Catalyst	Additive	Temperature (ºC)	Solvent	Time (h)	Yield/Selectivity (%)	Ref.
1[a]	CuI@UiO-67-IM (2 mol%, 1.7 mol% Cu)	-	80	H_2O	8	68[b]	***Inorg. Chem.*, 2017, 56, 8341-834**
2	CuI 1-D polymeric coordination complex (0.5 mg, 0.1 mol%)	-	50	CH_3CN/ H_2O	3.5	95	***Tetrahedron Lett.*,** 2018, 59, 2541-2545
3	$CuSO_4$•$5H_2O$ (9.4 mg)	Na ascorbate, *β*-cyclodextrin	r.t	H_2O	25 min	98	***J. Org. Chem.*,** 2012, 77, 4117-4122
4	Self- assembled poly(imidazole-acrylamide) Cu (0.25 mol%, 3 mg)	Na ascorbate	50	*t*-BuOH/H_2O	2.5	97	***J. Am. Chem. Soc.*,** 2012, 134, 9285-9290
5	Cu-tetracatechol metallopolymer (10 mg, 10 mol%)	-	50	*t*-BuOH/H_2O	2.5	99[b]	***Chem. Commun.*,** 2020, 56, 13044-13047
6	$CsCu_2I_3$@PCN-222(Fe) (10 mg)	-	r.t.	H_2O	~30 min	98	**This work**

[a] Consists of two steps

[b] Conversion

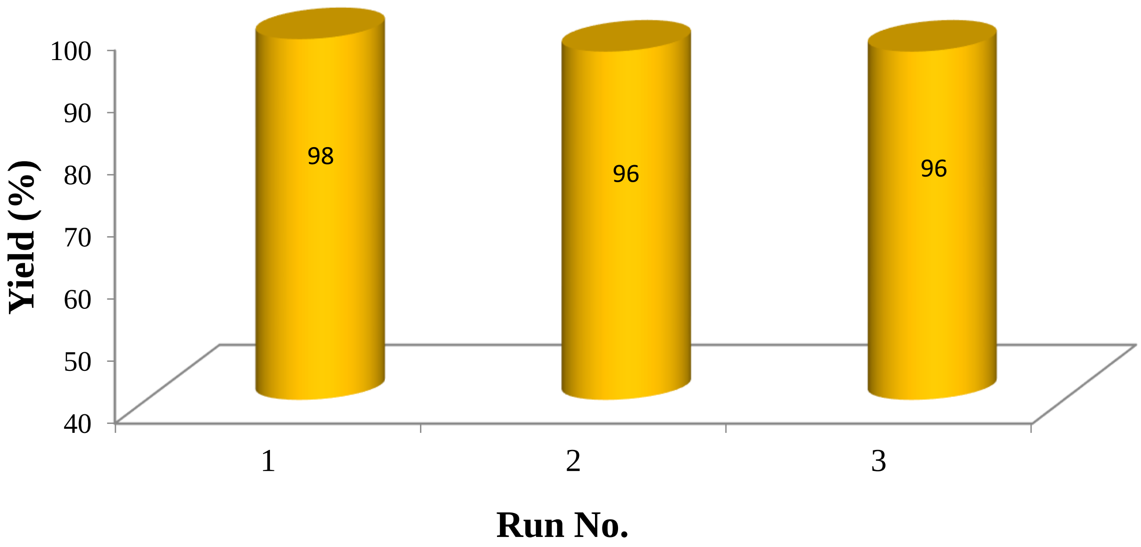

Figure S9. The recycle tests of $CsCu_2I_3$@PCN-222(Fe) for the click reaction.

Table S4. Comparison of tandem oxidation/Knoevenagel condensation reaction between benzyl alcohol and malononitrile catalyzed by $CsCu_2I_3$@PCN-222(Fe) and the other reported (photo)catalysts.

Entry	Catalyst	Condition	Solvent	Time (h)	Con. (%)	Yield (%)	Ref.
**1[a]**	g-C_3N_4/$FeWO_4$ (40 mg)	Visible-light irradiation (250 W Mercury lamp), under O_2 atmosphere	CH_3CN	14	84	75	***ACS Appl. Nano Mater.***, 2020, 3(7), 7057-7065
**2[a]**	NH_2-MIL-101(Fe) (20 mg)	Visible light (300 W Xe), O_2 (1 atm)	$C_6H_5CF_3$/CH_3CN	40	88	72	***Catal. Sci. Technol.***, 2015, 5, 1623-1628
**3[a]**	Au(III)@Cu(II)-MOF (13 mg)	Frist step: air, 110 °C; second step: r.t	Frist step: toluene, step second: toluene/methanol	23	99	>99	***Inorg. Chem.***, 2016, 55(13), 6685-6691
**4[b]**	Cu_3TATAT-3 (~68 mg)	TEMPO, 75 °C, O_2 (1 atm)	CH_3CN	12	95	99	***Dalton Trans***., 2016, 45(35), 13917-13924
**5**	Zr-MOF-NH_2 (100 mg)	UV-light irradiation, 90 °C	*p*-xylene	48	100	91	***Catal. Sci. Technol.***, 2014, 4, 625-628
**6**	$CsCu_2I_3$@PCN-222(Fe)(15 mg)	Visible LED light (33 ± 2 °C)	CH_3CN	15	100	96	**This work**

[a] Consists of two consecutive steps.

[b] Cu(II)/amine bifunctional, basic metal–organic framework (MOF)

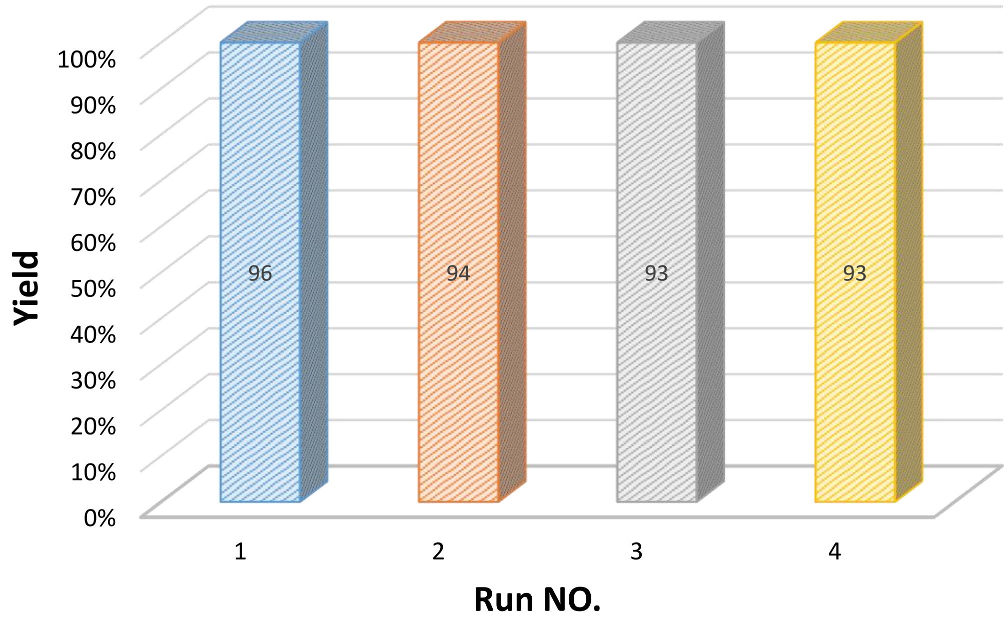

Figure S10. The recycle tests of $CsCu_2I_3$@PCN-222(Fe) photocatalyst for one-pot tandem selective photo-oxidation/Knoevenagel condensation reaction between benzyl alcohol and malononitrile.

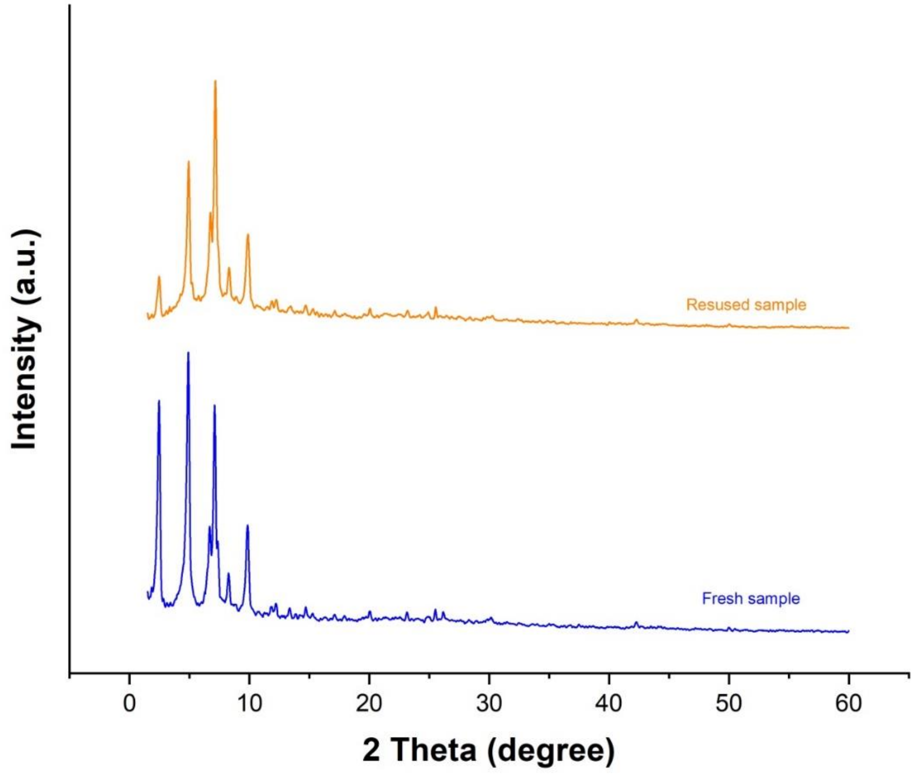

Figures S11. PXRD profiles of fresh and reused $CsCu_2I_3$@PCN-222(Fe) photocatalyst.

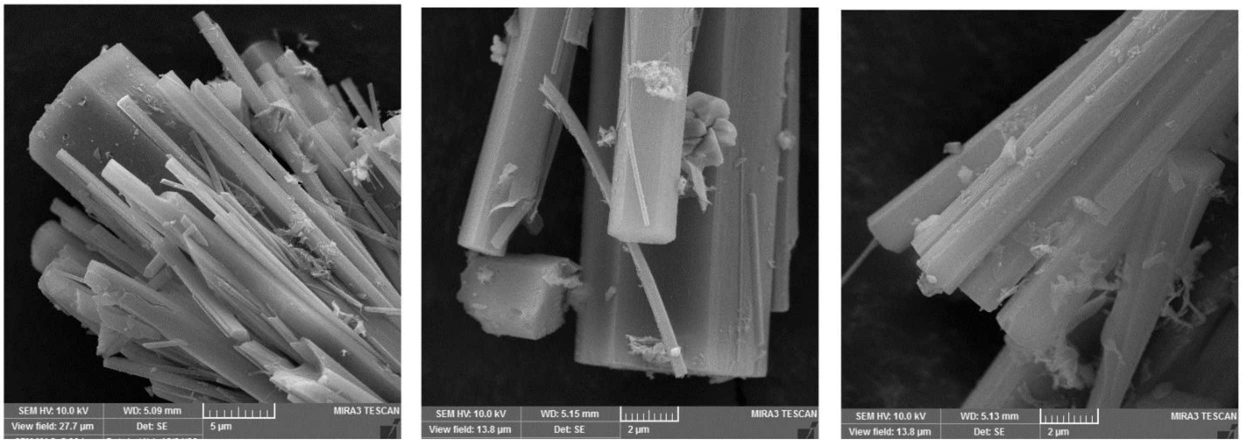

Figure S12. SEM images of reused $CsCu_2I_3$@PCN-222(Fe) photocatalyst.

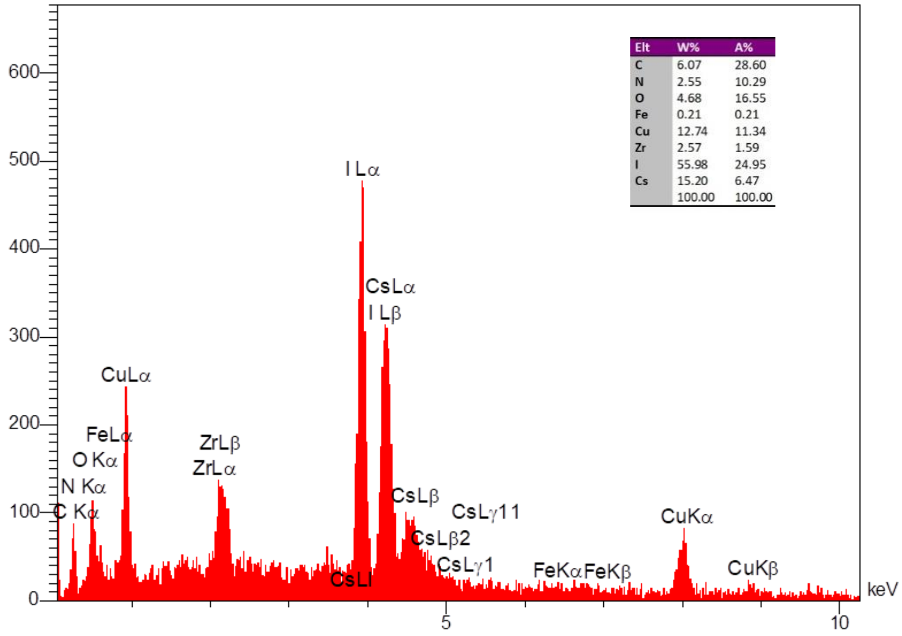

Figure S13. EDS elemental analysis of reused $CsCu_2I_3$@PCN-222(Fe) photocatalyst.

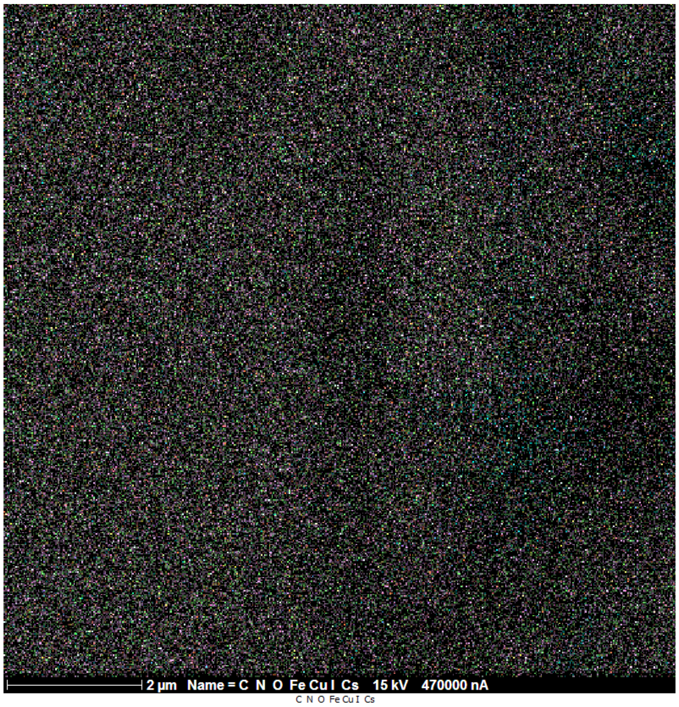

Figure S14. Mapping elemental analysis of reused $CsCu_2I_3$@PCN-222(Fe) photocatalyst.

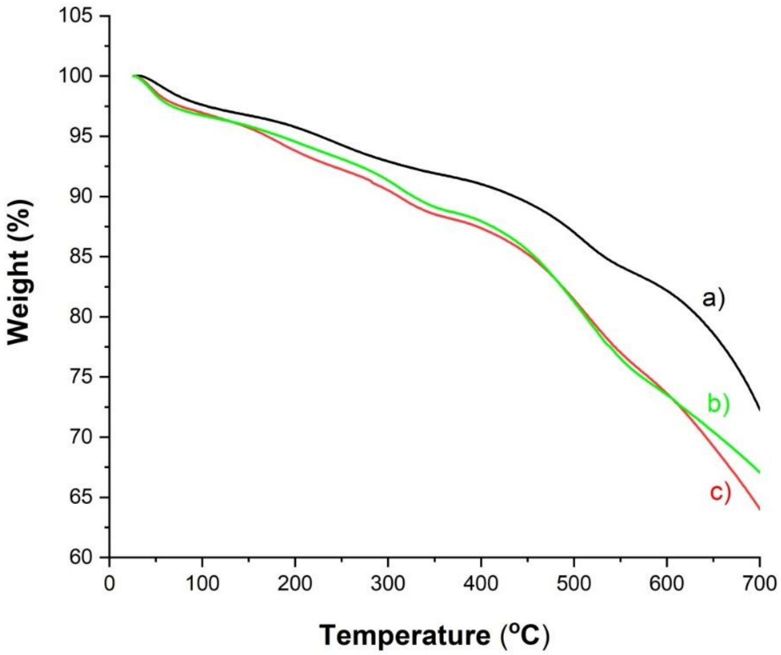

Figure S15. TGA profiles of a) fresh PCN-222(Fe), b) fresh and c) reused $CsCu_2I_3$@PCN-222(Fe) photocatalyst.

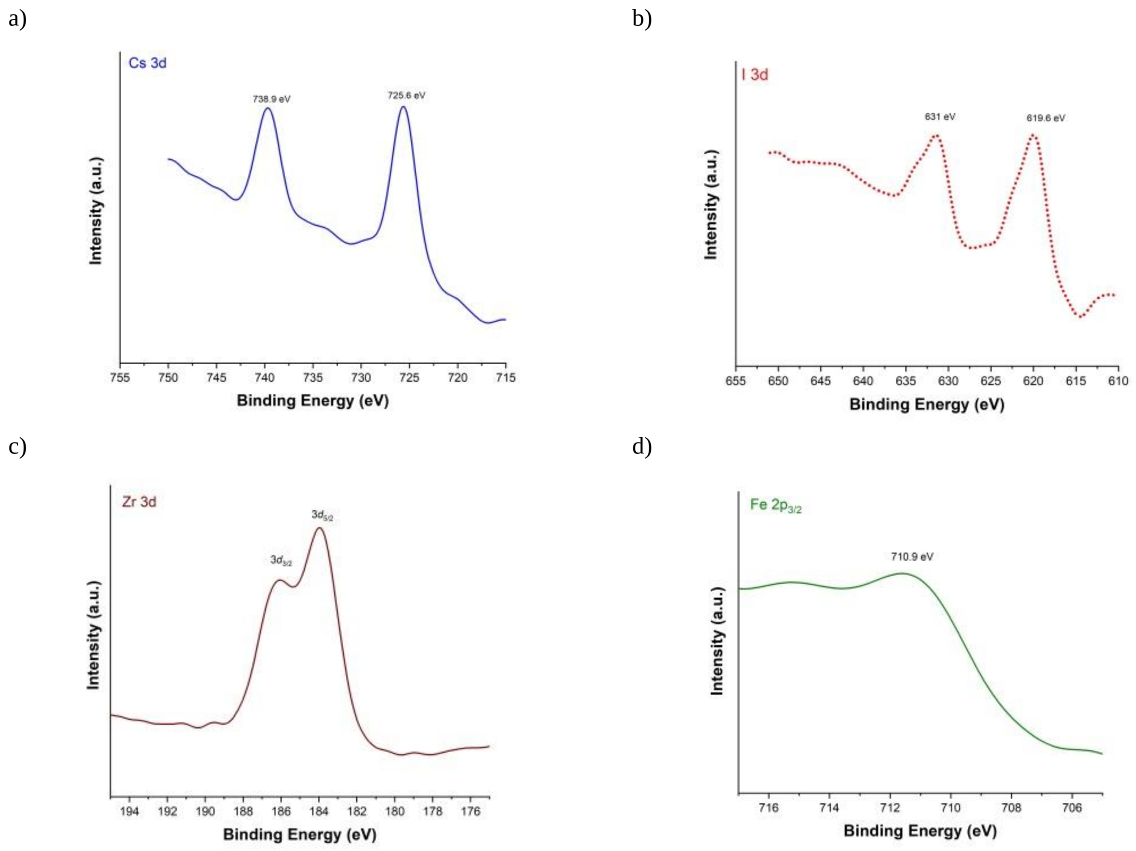

Figure S16. XPS spectrum of the $CsCu_2I_3$@PCN-222(Fe) with views of elements; a) Cs 3d, b) I 3d, c) Zr 3d, and d) Fe $2p_{3/2}$.

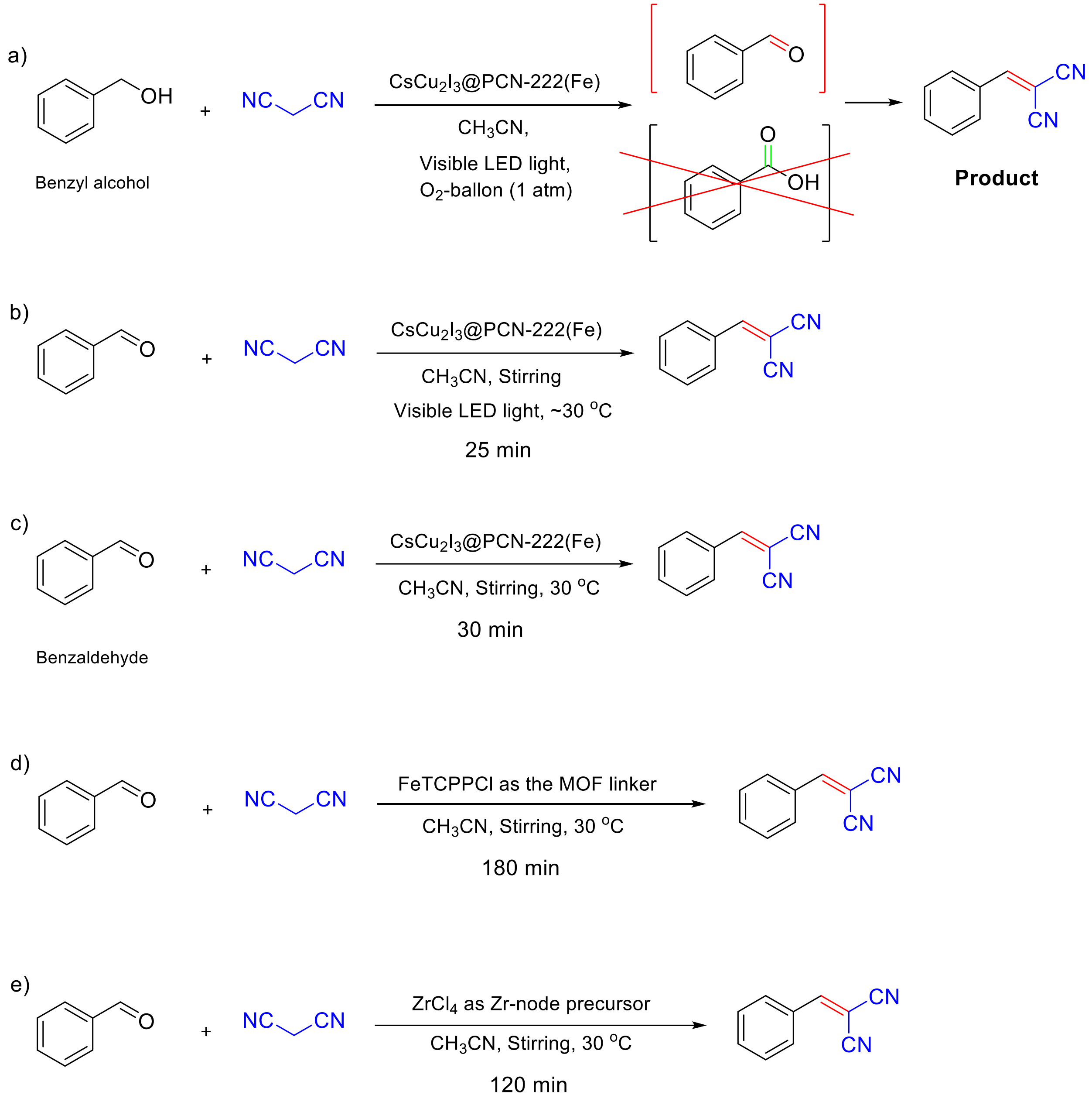

Scheme S1. Control experiments for the clarification of the reaction mechanism.

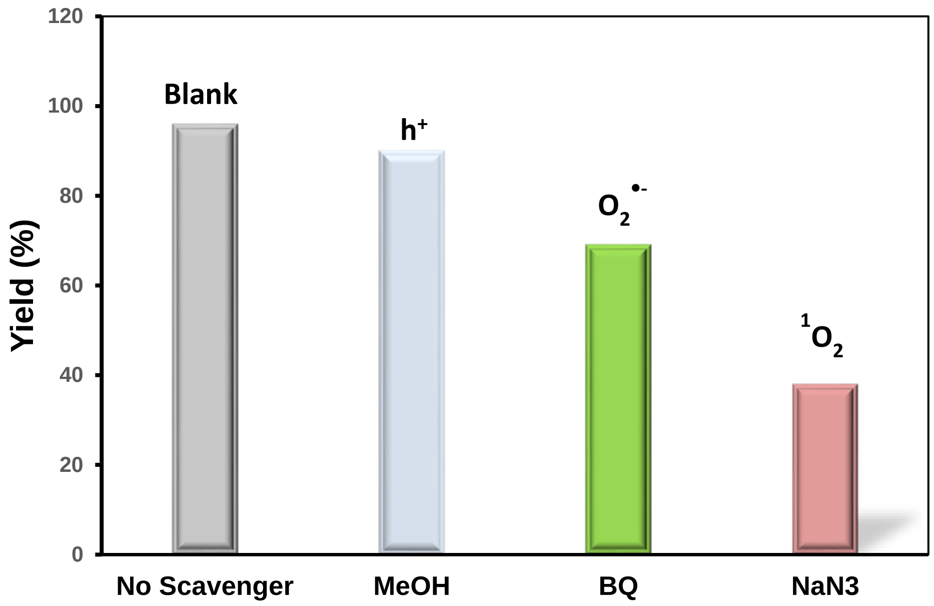

Figures S17. Inhibition experiments by using the quenchers.

Characterization data of the products:

1-Benzyl-4-phenyl-1H-1,2,3-triazole:^{1}H-NMR (300 MHz, $CDCl_3$): δ (ppm): 7.75-7.72 (m, 2H, -C_6H_5), 7.59 (s, 1H, -C_2HN_3), 7.36-7.19 (m, 8H, -C_6H_5), 5.51 (s, 2H, -CH_2); ^{13}C-NMR (75 MHz, $CDCl_3$) δ (ppm):148.38, 134.83, 130.69, 129.30, 128.93, 128.29, 128.20, 125.84, 119.59, 54.38.

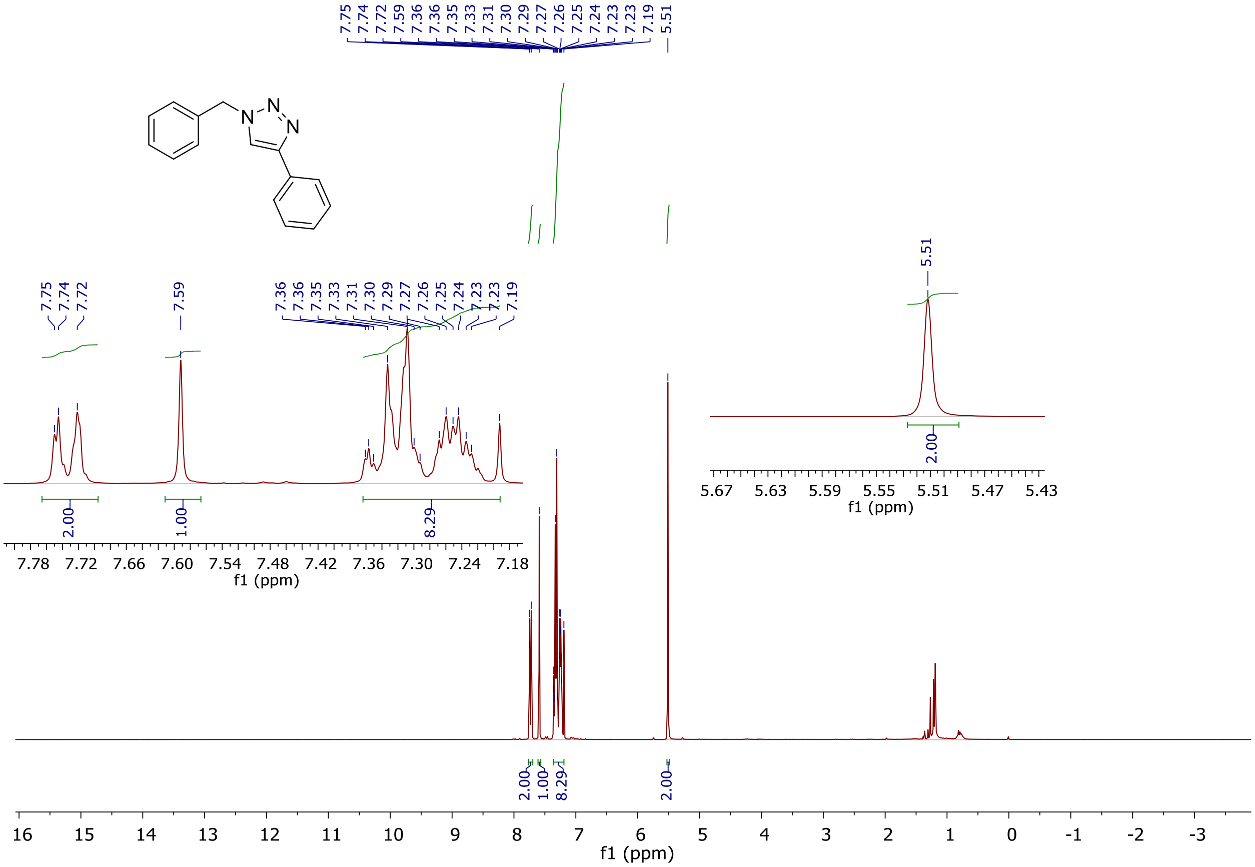

Figures S18. ^{1}H NMR spectrum of 1-benzyl-4-phenyl-1H-1,2,3-triazole.

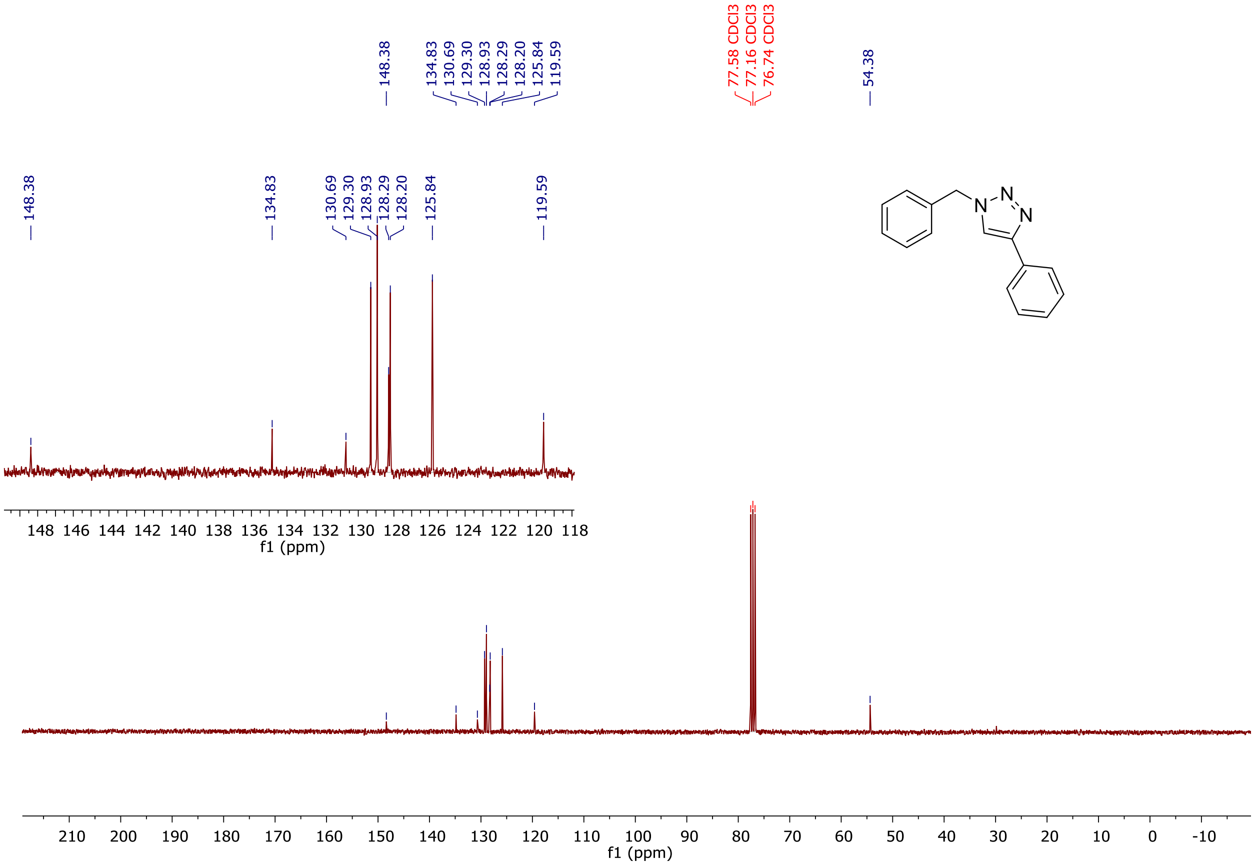

Figures S19. ^{13}C NMR spectrum of 1-benzyl-4-phenyl-1H-1,2,3-triazole.

2-Benzylidenemalononitrile: m.p.: 83-85 ˚C [82-83][S7]; ^{1}H NMR (300 MHz, DMSO-d_6) δ (ppm): 8.56 (s, 1H), 7.97 (d, J = 6 Hz, 2H), 7.61-7.74 (m, 3H); ^{13}C NMR (75 MHz, DMSO-d_6): δ = 162.05, 134.87, 131.76, 130.98, 130.01, 114.68, 113.69, 82.08.

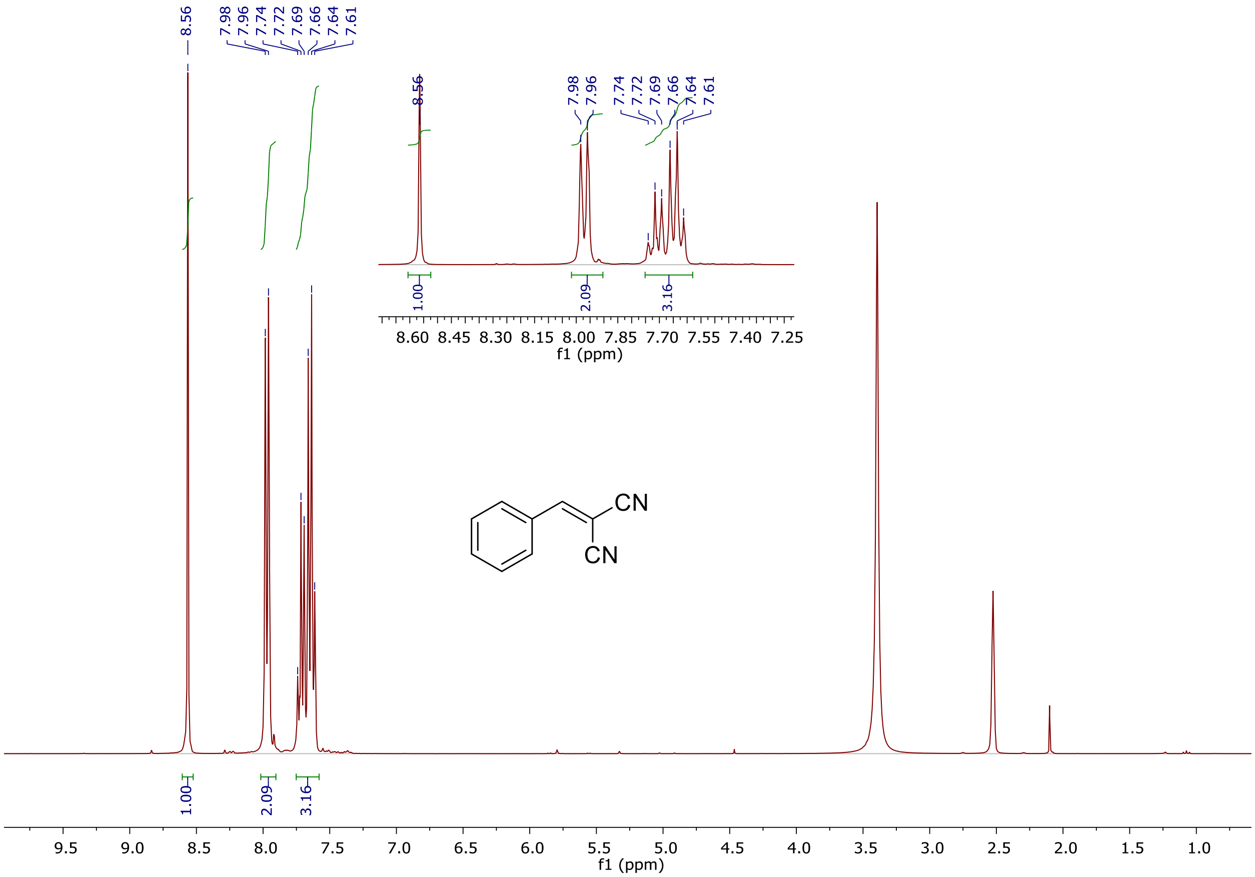

Figures S20. ^{1}H NMR spectrum of 2-benzylidenemalononitrile.

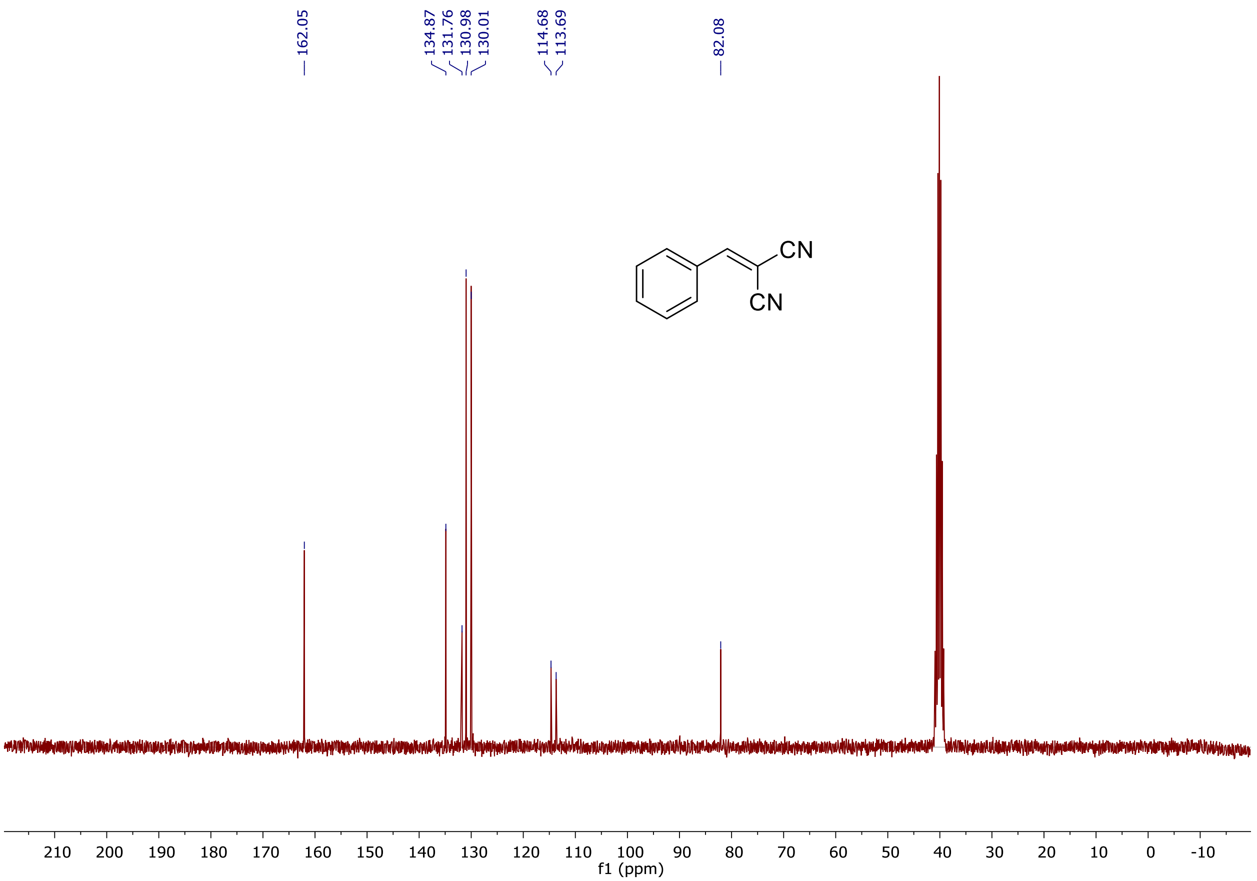

Figures S21. ^{13}C NMR spectrum of 2-benzylidenemalononitrile.

2-(4-Methylbenzylidene)malononitrile: m.p.:124-126 ˚C [123-126][S8]; ^{1}H-NMR (300 MHz, DMSO-d_6): δ (ppm): 8.47 (s, 1H), 7.87 (d, J = 6 Hz, 2H), 7.01 (d, J = 6 Hz, 2H), 2.42 (s, 3H); ^{13}C-NMR (75 MHz, DMSO-d_6) δ (ppm): 161.73, 146.16, 131.17, 130.62, 129.21, 114.87, 113.91, 80.35, 21.94.

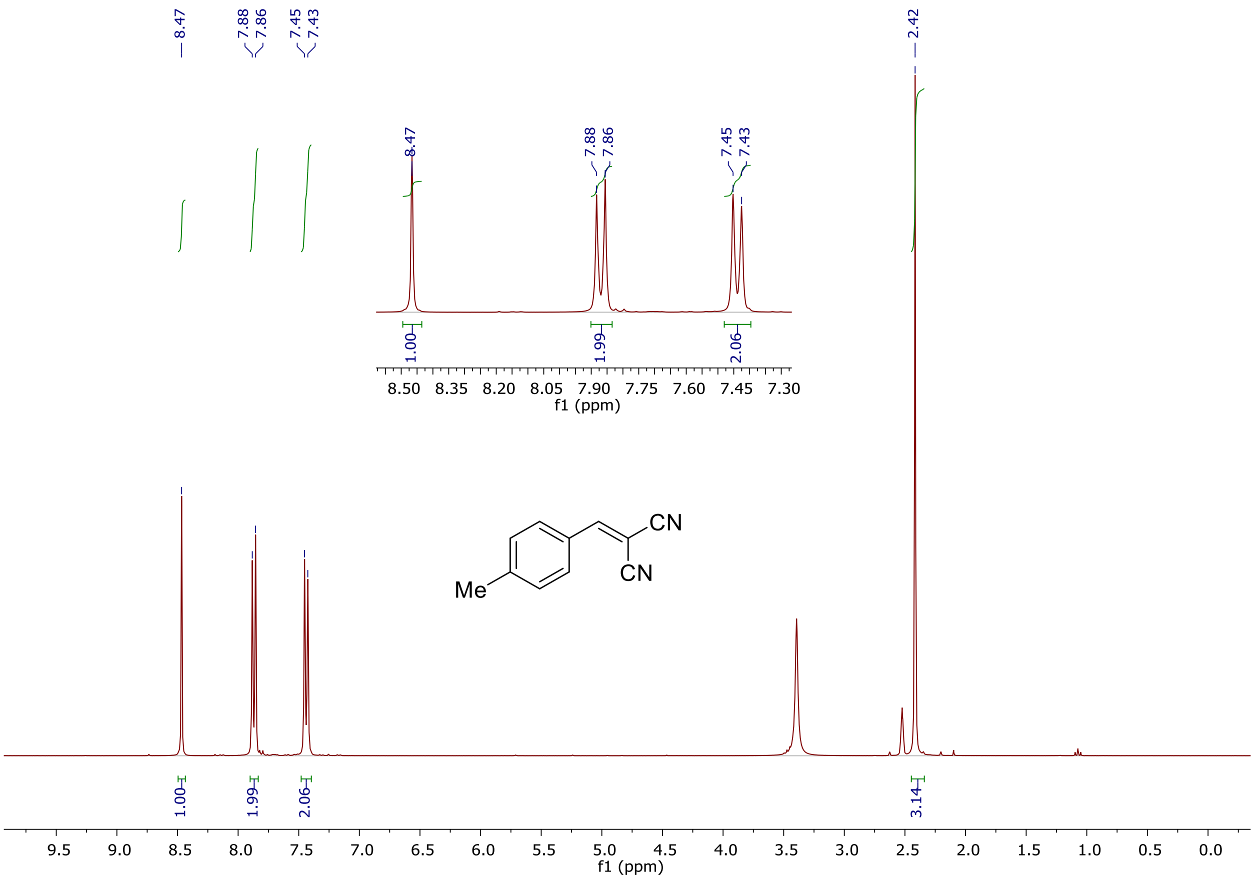

Figures S22. ^{1}H NMR spectrum of 2-(4-methylbenzylidene)malononitrile.

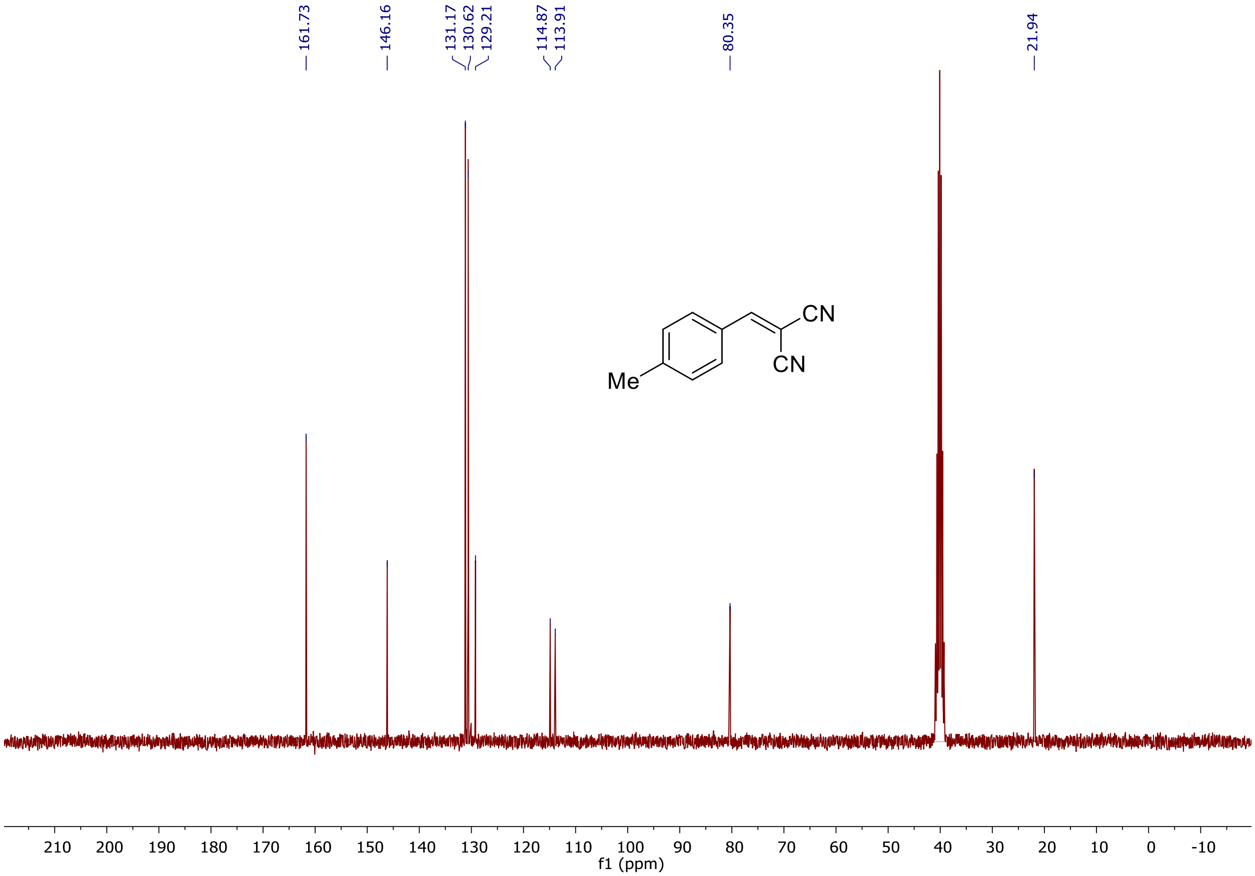

Figures S23. ^{13}C NMR spectrum of 2-(4-methylbenzylidene)malononitrile.

2-(4-Chlorobenzylidene)malononitrile: m.p.:163-165 ˚C [162-164][S7];^{1}H-NMR (300 MHz, DMSO-d_6): δ (ppm): 8.85 (s, 1H), 7.96 (d, J = 8.5 Hz, 2H), 7.72 (d, J = 8.5 Hz, 2H),; ^{13}C-NMR (75 MHz, DMSO-d_6) δ (ppm): 160.58, 139.53, 132.61, 130.54, 130.19, 114.52, 113.48, 82.70.

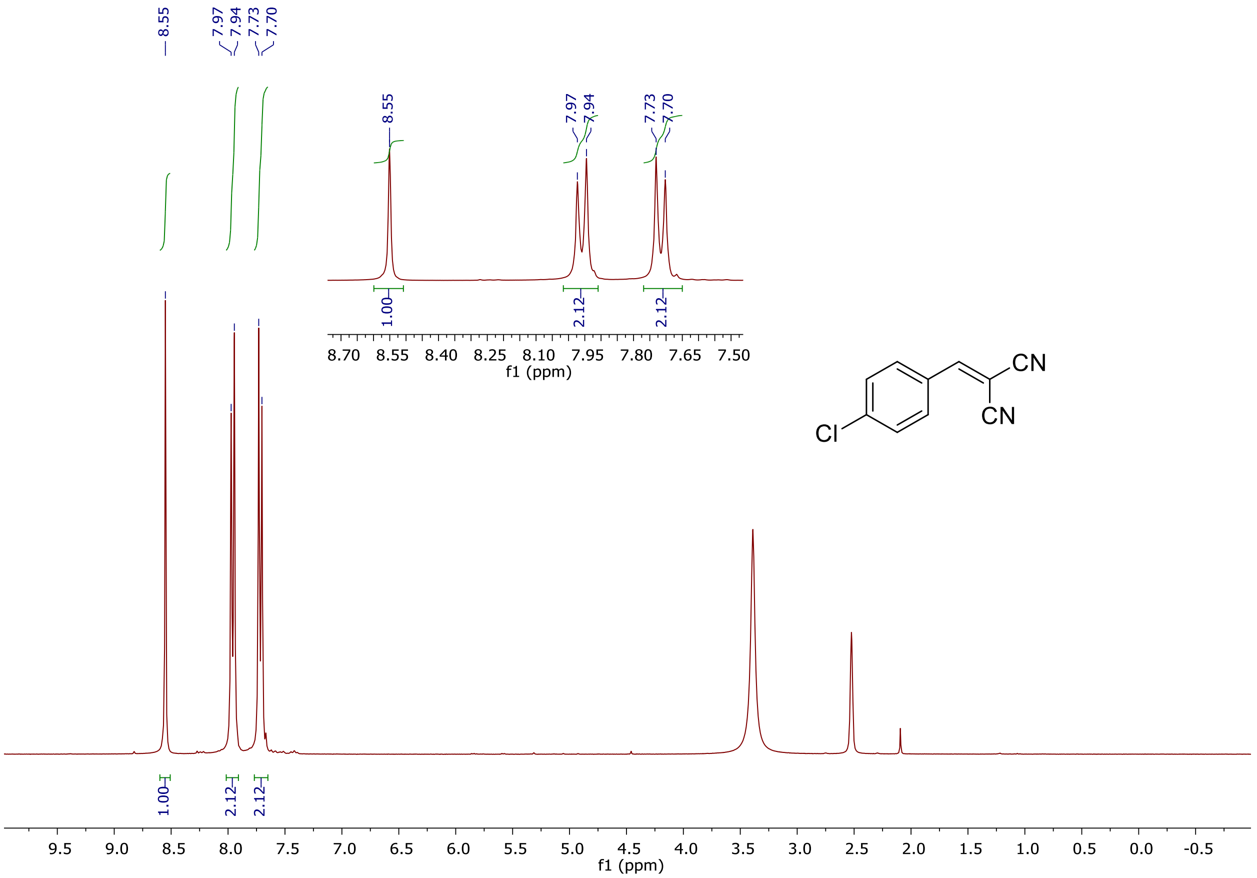

Figures S24. ^{1}H NMR spectrum of 2-(4-chlorobenzylidene)malononitrile.

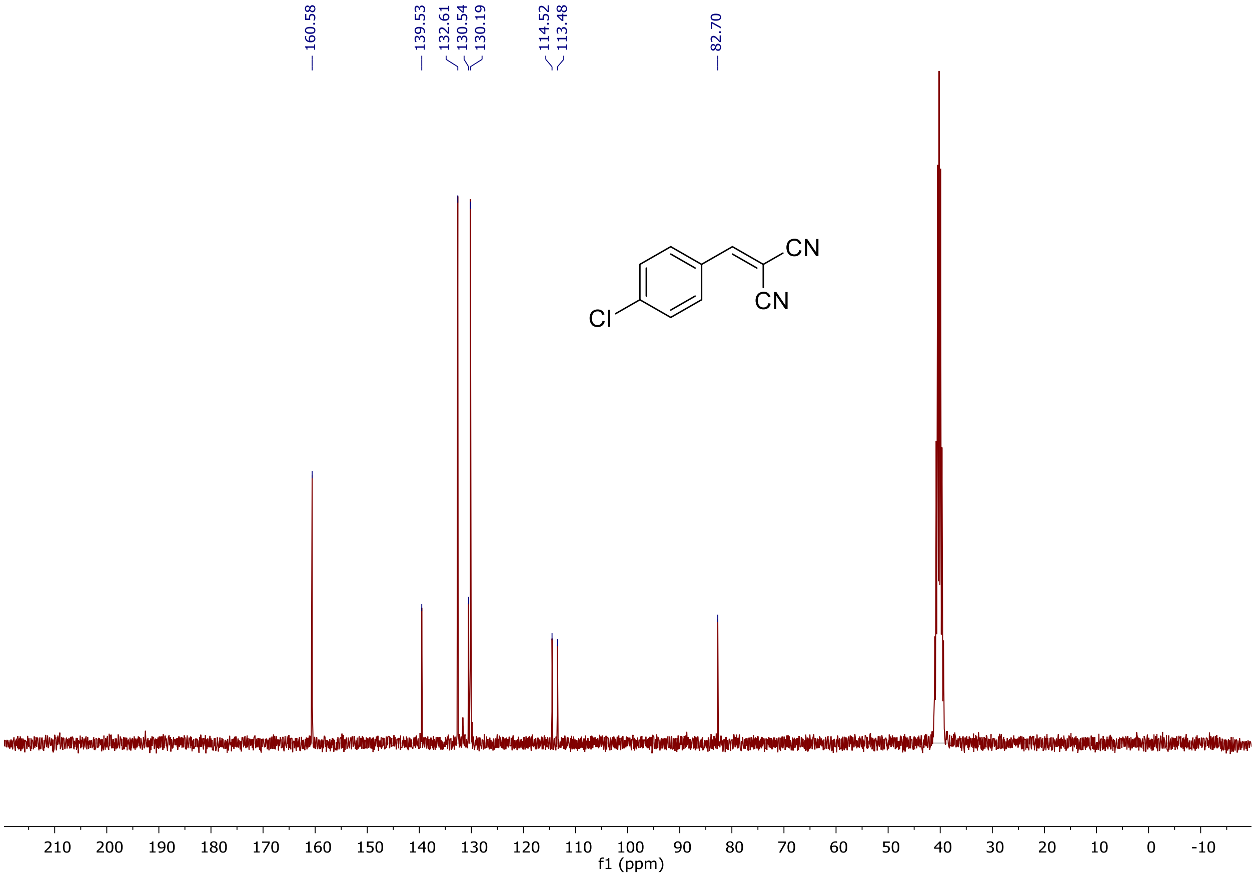

Figures S25. ^{13}C NMR spectrum of 2-(4-chlorobenzylidene)malononitrile.

References:

S1. Zhang, C.; Li, H.; Li, C.; Li, Z. *Molecules*, **2020**, 25, 168.

S2. Wu, S.-c.; You, X.; Yang, C.; Cheng, J.-h. *Water Sci. Technol*., **2017**, 75, 2800.

S3. Yang, J.-M.; Yang, B.-C.; Zhang, Y.; Yang, R.-N.; Ji, S.-S.; Wang, Q.; Quan, S.; Zhang, R.-Z. *Micro. Meso. Mater*., **2020**, 292, 109764.

S4. Yang, J.-M.; Ying, R.-J.; Han, C.-X.; Hu, Q.-T.; Xu, H.-M.; Li, J.-H.; Wang, Q.; Zhang, W. *Dalton Trans*., **2018**, 47, 3913.

S5. Li, H.; Cao, X.; Zhang, C.; Yu, Q.; Zhao, Z.; Niu, X.; Sun, X.; Liu, Y.; Ma, L.; Li, Z. *RSC Adv*., **2017**, 7, 16273.

S6. Tong, M.; Liu, D.; Yang, Q.; Devautour-Vinot, S.; Maurin, G.; Zhong, C. *J. Mater. Chem. A*, **2013**, 1, 8534.

S7. R. Khoshnavazi, L. Bahrami, F. Havasi, *RSC Adv*., **2016**, 6, 100962.

S8. H. Ghafuri, G. Jafari, A. Rashidizadeh, F. Manteghi, *Mol. Catal.*, **2019**, 475, 110491.
